# A bundled anti-parallel cytochrome nanowire structure suggests roles in cell-cell electron transfer and biofilm formation

**DOI:** 10.1128/mbio.00906-26

**Published:** 2026-08-26

**Authors:** Holly A. Petersen, Chi H. Chan, Gilbert O. Carpenter, Media Zakizadeh Tabari, Jessie Lynda Fields, Ayisha Zia, Shane T. Rich-New, Allon I. Hochbaum, Daniel R. Bond, Fengbin Wang

**Affiliations:** 1 Department of Biochemistry and Molecular Genetics, University of Alabama at Birmingham, Birmingham, Alabama, USA; 2 Department of Plant and, Microbial Biology, BioTechnology Institute, University of Minnesota, Saint Paul, Minnesota, USA; 3 Materials Science and Engineering, University of California Irvine 685364, Irvine, California, USA; 4 Department of Chemical and Biomolecular Engineering University of California Irvine 542560 , Irvine, California, USA; 5 Department of Chemistry, University of California Irvine 8788, Irvine, California, USA; 6 Department of Molecular Biology and Biochemistry, University of California Irvine, Irvine, California, USA; 7 Gregory Fleming James Cystic Fibrosis Research Center, University of Alabama at Birmingham, Birmingham, Alabama, USA; Virginia Polytechnic Institute and State University, Blacksburg, Virginia, USA

**Keywords:** cytochrome nanowires, microbial nanowires, helical filament, extracellular electron transfer, biofilm formation, pili bundles, cryo-EM

## Abstract

Long-range extracellular electron transfer enables respiring microbes to use minerals, organisms, or electrodes as electron acceptors by transporting electrons microns away from the cell surface. This process is primarily studied in *Geobacter sulfurreducens*, which produces at least three distinct, micrometer-long, multiheme cytochrome nanowires capable of electron transfer. However, the distribution and higher-order structure of such nanowires remains largely unknown. Here, we employed cryo-electron microscopy to determine the atomic structure of a unique cytochrome nanowire from *Desulfuromonas soudanensis* WTL, a halophilic, iron- and electrode-reducing bacterium from deep subsurface brine. These filaments are based on an OmcE tetraheme cytochrome homolog that assembles into highly ordered bundles of anti-parallel filaments. Electronic measurements of purified nanowire bundle films reveal that these bundles are conductive, with conductance increasing in a concentration-dependent manner. The apparent midpoint potential of these OmcE bundles was −145 mV vs SHE. They exhibit redox activity over a ~370 mV window attributable to five electrostatically coupled hemes, suggesting heme–heme interaction beyond the boundaries of individual OmcE repeat units. Observations of these bundles under diverse buffer conditions suggest that they represent stable supramolecular assemblies, although direct *in vivo* confirmation within intact biofilms remains a future goal. Furthermore, a similar cytochrome bundle structure was observed in *Geobacter metallireducens*, showing that this quaternary structure may be a common feature among nanowires secreted by electroactive microbes. Our findings demonstrate that cytochrome nanowires are widespread and can form specialized bundle interfaces. This novel state could facilitate conductive biofilm formation and form the basis for direct microbial electron exchange.

**IMPORTANCE:**

All available structures of filaments containing a central core of closely spaced multiheme c-type cytochromes are derived from only a few organisms and are based on individual fibers easily imaged by cryo-electron microscopy. While imaging enriched preparations of extracellular appendages from a bacterium isolated from deep subsurface brine, we observed ordered bundles of anti-parallel cytochrome filaments. This quaternary arrangement contained specific contacts between adjacent nanowires, and a similar structure could be found in a relative from a related genus. This first report of bundling by nanowires suggests that such conductive structures may be missed in traditional surveys searching for novel filaments and provides an explanation for how nanowires could directly link adjacent cells in biofilms and granular communities.

## INTRODUCTION

In environments lacking oxygen, diverse obligately anaerobic and facultative prokaryotes rely on extracellular electron transfer to move electrons beyond their outer membrane to a terminal electron acceptor (1, 2). First observed in the reduction of iron and manganese oxides (3, 4), extracellular electron transfer allows cells to also maintain redox homeostasis via interspecies electron transfer to nearby syntrophic partners such as methanogens (5, 6). These organisms still generate ATP through electron transport across the cytoplasmic membrane but must direct electrons from the quinone pool to periplasmic carriers and ultimately across the insulating outer membrane to reach insoluble extracellular chelated minerals, oxide particles, distal organisms, and surfaces (7–9). Some organisms rely primarily on diffusive redox shuttles such as flavins to carry electrons from outer membrane multiheme cytochromes, as observed in *Shewanella* (10–12). However, if soluble metals or electron shuttles are not readily available, redox proteins must physically contact the acceptor surfaces, which can be microns away (13–15). To accomplish this, organisms such as *Geobacter sulfurreducens* (16–21) produce extracellular helical conductive appendages.

*G. sulfurreducens* has been used as a model system for interrogating the molecular machinery of bacterial extracellular electron transfer (22–24). Initial analysis of the genome revealed a massive metabolic investment of multiheme cytochrome genes, including 70+ open reading frames with three or more CxxCH heme *c*-binding motifs. For decades, these cytochromes were hypothesized to contribute to electron transfer only in the inner membrane, periplasm, and outer membrane until cryogenic electron microscopy (cryo-EM) revealed that at least three different multiheme *c*-type cytochromes in *G. sulfurreducens* polymerize into micrometer-long appendages: OmcS (19, 21), OmcE (17), and OmcZ (18, 20). Although these three proteins possess minimal secondary structure and share little structural or sequence similarity with each other, they can be classified into two functional archetypes based on the arrangement of hemes within the assembled cytochrome filaments: “insulated” and “leaky” nanowires (20, 25). OmcS and OmcE have their heme molecules well insulated by surrounding protein backbones. This type of insulated cytochrome nanowire, with identical heme arrangements but completely different subunit structure, has also been observed in Archaea (26), such as *Pyrobaculum calidifontis* and *Archaeoglobus veneficus*.

In contrast, OmcZ has a significantly different heme arrangement. Each subunit contains a branched, solvent-exposed “leaky” heme in addition to the linear conductive chain of hemes (18). Without this exposed heme, the linear chain would be insulated, resembling OmcS and OmcE. As the *omcZ* gene is upregulated by 400–800% in electrode-associated biofilms (27, 28), and its deletion impairs anodic electron transfer to electrodes (28–30), networks of OmcZ filaments are proposed to form an electron grid that makes a conductive biofilm matrix, providing multiple pathways for electron flow (18). Cryo-EM visualization of sheared OmcZ does reveal many nanowires aggregated together, but without defined quaternary, repeating interactions (18, 20). To date, no atomic structures showing direct repeated interactions between any class of nanowire have been reported.

Microbial appendages are common components of biofilms, along with other scaffolding biomaterials such as exopolysaccharides and extracellular DNA (31). In fact, appendages often dictate biofilm formation (32–34). For example, retractable bacterial type IV pili frequently play a critical role for adherence to surfaces, ultimately leading to colonization and biofilm formation (35). Appendage-driven biofilm formation can also involve formation of specific pilus-to-pilus interfaces, resulting in the formation of ordered pili bundles. One such bundle structure was resolved by cryo-EM in *Pyrobaculum calidifontis* (36), where the pili assemble into highly ordered bipolar bundles. The bipolar nature of these bundles likely arises from the association of pili from at least two different cells. Pili and secreted proteins important for bacterial biofilm formation have also been observed to form bundles, such as secreted TasA (37) in *Bacillus subtilis* and the chaperone–usher pathway (CUP) pilus CupE (38) in *Pseudomonas aeruginosa*. However, only single-filament structures were resolved in these cases; therefore, the pilus-pilus interfaces within the bundles, whether specific or not, remain unresolved. More recently, anti-parallel bundles made up of archaic CUP pili have been described by cryo-EM. These form geometrically distinct pilus “stacks” in biofilms, providing an architecture that allows cells to tether together (39, 40).

In this work, we present the cryo-EM structure of a bundle formed by cytochrome nanowires produced by *Desulfuromonas soudanensis* WTL, a halophilic iron-reducing bacterium isolated from anoxic deep subsurface brine. This is the first structure of a cytochrome nanowire in a bacterium outside of *G. sulfurreducens*. We show that the primary cytochrome building block is homologous to *G. sulfurreducens* OmcE. However, our structural data clearly reveal that *D. soudanensis* OmcE nanowires form anti-parallel bundles mediated by two types of filament-to-filament interactions. To confirm the electronic functionality of these OmcE assemblies, we performed current–voltage (I–V) measurements on purified WTL fiber films, which demonstrated concentration-dependent conductance that significantly exceeded that of the buffer controls. In-depth sequence analysis identified likely OmcE homologs in five different bacterial phyla and revealed highly variable sequence regions within the OmcE subunit.

We further demonstrate that this bundling phenomenon is not unique, showing that OmcE nanowires also form bundles in *Geobacter metallireducens*, a bacterium evolutionarily much closer to *G. sulfurreducens*. Such large, ordered bundles of nanowires are of great interest in synthetic biology and bio-nanotechnology, offering potential biosensing applications such as detection of viruses and tumor biomarkers (41–43). Furthermore, by combining this with structural analysis of heme arrays from over 500 AlphaFold (44) predicted OmcE-like proteins, we provide insights into how such architecture might facilitate community-level electron flux and multicellular cooperation within these dual-function cytochrome nanowire bundles.

## RESULTS

### *D. soudanensis* WTL produces conductive bundles made of OmcE nanowires

**Fig 1:**
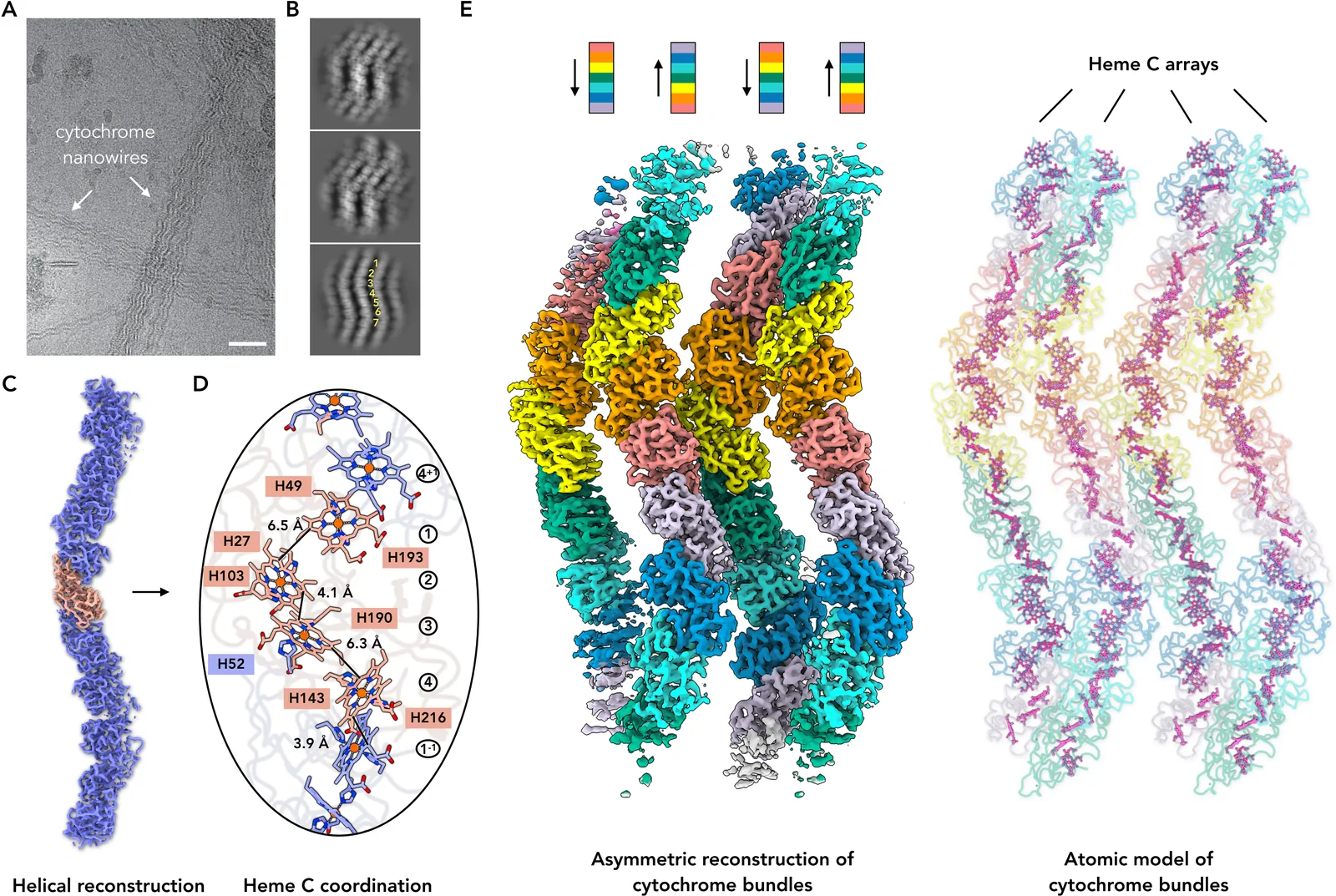
*D. soudanensis* WTL produces bundles of OmcE cytochrome nanowires. (**A**) Representative cryo-EM micrograph of cytochrome bundles from *D. soudanensis* WTL. Scale bar: 50 nm. (**B**) 2D class averages of cytochrome bundles. (**C**) Helical reconstruction of a single cytochrome filament within the cytochrome bundles, with one cytochrome subunit highlighted in salmon. (**D**) Arrangement of heme molecules within the helical reconstruction. (**E**) Asymmetric reconstruction of cytochrome bundles and the corresponding atomic model built into the map. To clearly demonstrate the filament’s polarity, seven different subunit conformations are colored using a rainbow scheme, as indicated. The protein backbone of the atomic model is colored to match the map, while all heme molecules are colored magenta.

In order to investigate *D. soudanensis* WTL extracellular appendages, we grew 1 L of *D. soudanensis* WTL cells (45) and enriched extracellular filaments by ammonium sulfate precipitation as previously described (46). Cryo-EM micrographs of the enriched extracellular filaments showed that they formed filament bundles with diameters up to 150 nm, while single filaments were sparsely observed (Fig. 1A). To validate that the bundle formation was physiologically relevant and not introduced by the enrichment buffer, we examined sheared filaments under two other conditions: phosphate-buffered saline and the bacterial growth medium (45) (containing a total concentration of over 500 mM of various salts that mimic the natural brine, including CaCl_2_, MgCl_2_, and NaCl). Strikingly, filamentous bundles were observed under all tested conditions, and single filaments were rarely seen (Fig. S1), suggesting that the sheared appendages interact and bundle together regardless of salinity. However, because these observations are based on images of sheared and purified nanowires rather than on nanowires within intact, native biofilms, we cannot entirely rule out the possibility that some degree of bundle formation or aggregation occurs during sample manipulation and purification. Consequently, direct *in vivo* verification via cryo-electron tomography (cryo-ET) of native biofilms remains a key objective for future studies.

After data collection, the resulting 2D averages revealed only seven distinct views along the filament (Fig. 1B), suggesting a preferred orientation problem, a rare occurrence in helical reconstructions. Indexing the power spectrum of the bundles indicated a helical twist of ~51.4 degrees and a rise of ~30.8 Å (Fig. S2), confirming the observations in the 2D classes. To obtain additional views of the filaments, tilted data were collected with the sample grid tilted at −12°, +12°, and +4°, respectively (Fig. S3). With the inclusion of these tilted micrographs, helical reconstruction of the single cytochrome filament reached a resolution of 3.4 Å, as determined by map-to-map FSC (Fig. S2).

The cytochrome building block was identified as DSOUD_2954, a homolog of the tetraheme OmcE (17) first reported in *G. sulfurreducens*. Consistent with the structures of previously reported “insulated” cytochrome nanowires in *G. sulfurreducens* (17, 19) and two Archaeal species (26), the orientation between adjacent hemes was either stacked or T-shaped, with minimum distances between porphyrins ranging from 3.9 to 6.5 Å (Fig. 1D). On the surface of the *D. soudanensis* OmcE, two ion-binding sites were observed. Based on the coordinating distances (47) and medium components, these ions are likely to be calcium, which possibly stabilizes the cytochrome nanowires under high salt conditions (Fig. S4).

After helical averaging, only a single cytochrome fiber could be seen in the cryo-EM volume, suggesting that the bundle lacked global helical symmetry. We then asked the question of how individual cytochrome filaments interacted to form these large bundles. To avoid introducing reference bias, we used the aligned particles and volumes obtained after helical refinement to perform 3D classification with 10 classes. If multiple specific interfaces existed between single fibers within the bundle, these different classes could then be sorted out and reconstructed independently.

Following 3D classification, allowing these additional classes revealed new, ordered cytochrome filaments adjacent to the starting single-filament reference. Manual inspection of all these volumes consistently showed the same anti-parallel packing arrangement between cytochrome nanowires (Fig. 1E). This difference in polarity between adjacent filaments within the same bundle suggests that they likely originate from different bacterial cells, and the inter-filament interfaces are specific. These 3D classes were subsequently merged for an asymmetric reconstruction, which reached a global resolution of 3.1 Å as judged by map-to-map FSC. The local resolution varies from 2.6 to 5.0 Å, as determined by local resolution estimation (Fig. S5).

### *D. soudanensis* bundles are anti-parallel arrays of tightly packed cytochrome nanowires

**Fig 2:**
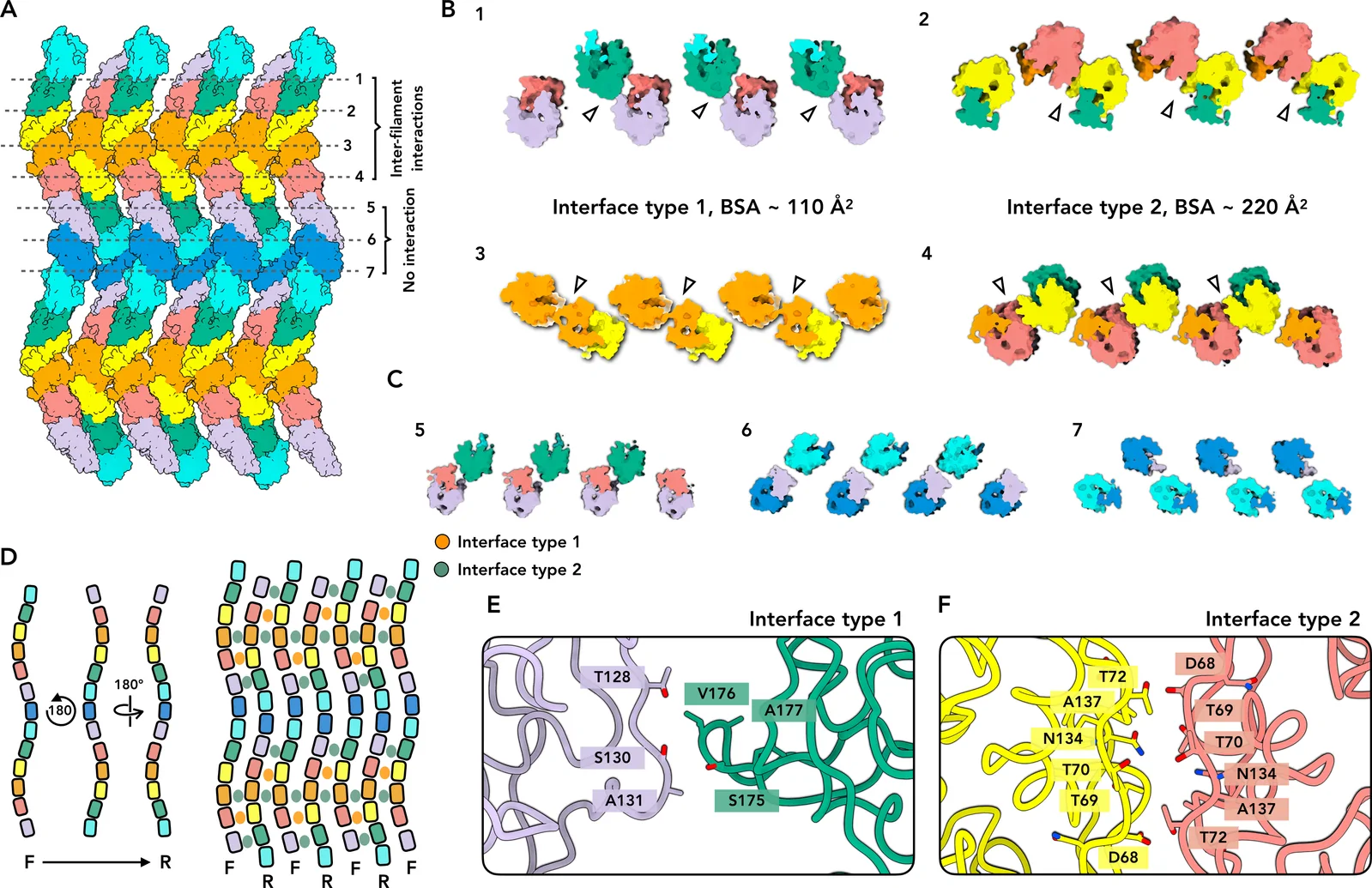
Binding interface within anti-parallel arrays of cytochrome nanowires. (**A**) Expanded atomic model of cytochrome arrays based on the obtained cryo-EM map. Different subunit conformations are colored using the same rainbow scheme as in Fig. 1. Seven unique inter-filament conformations are indicated by dashed lines. (**B and C**) The seven unique inter-filament conformations corresponding to those indicated in panel** A** are viewed from the top. Two types of interfaces are shown in panel **B**, while three conformations without evident inter-filament interfaces are shown in panel** C**. (**D**) A schematic illustrating the anti-parallel arrangement of cytochrome bundles and the locations where the interfaces are observed. (**E and F**) Zoomed-in views of the type 1 and type 2 interfaces.

Because the asymmetric reconstruction provided a high-resolution map of a large section of the bundle, we were able to use the known helical parameters of individual filaments to expand the atomic model and visualize the complete filament array (Fig. 2A). As indicated by the helical twist, 2D classification, and 3D reconstruction, the cytochrome subunit circles back to an identical environment every seven subunits (Fig. 1E and Fig. 2A). Furthermore, the 3D reconstruction revealed that every other cytochrome filament in the bundling array not only has the same polarity but is also in register (Fig. 2A). Therefore, to examine inter-filament interfaces, we only needed to consider relationships within these seven distinct cytochrome subunit states, using a single cytochrome filament as a reference. These seven states are visualized by drawing imaginary lines perpendicular to the bundle direction (Fig. 2A). Intriguingly, interfaces were observed for only four of these seven states (Fig. 2B); in states where filaments were sufficiently close to form detectable contacts, the amino acid configurations were different (Fig. 2C).

Where inter-filament contacts were detected, two unique interfaces were observed, with buried surface areas of 110 Å (2) (type 1 interface) and 220 Å (2) (type 2 interface), as estimated by PISA (48). Despite being small for an individual interface, these interfaces accumulate rapidly along the bundles (Fig. 2D). For instance, between two adjacent cytochrome filaments, the interface area is 440 Å (2) per 21.7 nm. This scales to a substantial interface of approximately 101,000 Å (2) for two 5 µm long cytochrome filaments, and this is amplified further in a bundling array containing dozens of such long filaments. When looking at the interfaces more closely, the type 1 interface is asymmetric, involving two loop regions: T128–A131 from one subunit and S175–A177 from another subunit (Fig. 2E). In contrast, the type 2 interface is symmetric, primarily mediated by the same loop regions, D68–T72 and N134–A137, between two subunits (Fig. 2F).

**Fig 3:**
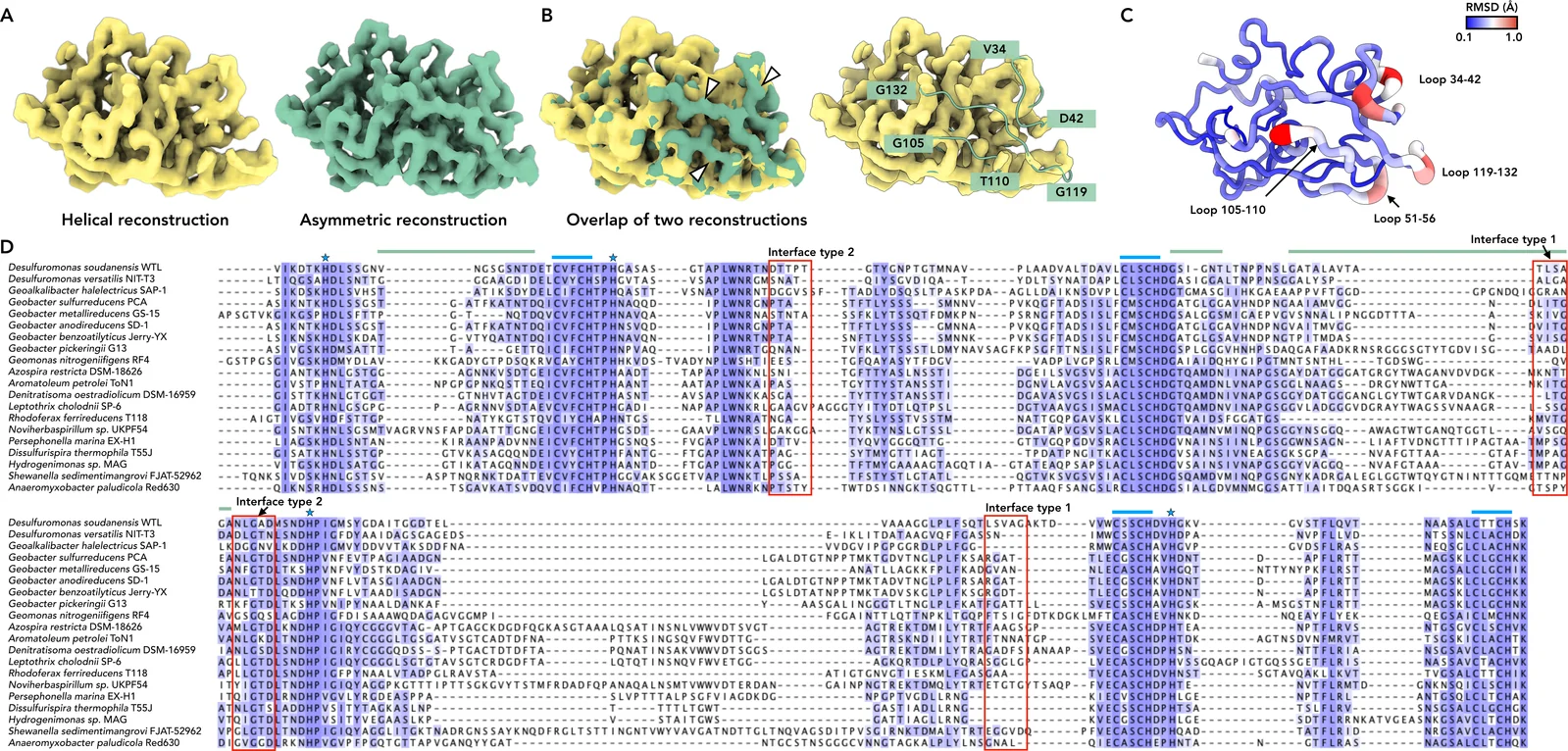
Asymmetric reconstruction recovers loop density involved in WTL bundling interface, which is not conserved among OmcE homologs. (**A**) Cryo-EM maps of a single *D. soudanensis* WTL OmcE monomer, obtained from helical refinement (left) and asymmetric refinement (right). (**B**) Overlay of the two volumes from panel **A** reveals that several loop regions in the helical refinement are smeared out when helical symmetry is applied, suggesting that they have different conformations. (**C**) RMSD analysis of seven different cytochrome conformations from asymmetric refinement indicates that similar loop regions have adopted slightly different conformations. (**D**) Structure-based sequence alignment of OmcE homologs from various bacteria. All selected OmcE sequences have an outer membrane porin encoded nearby on the chromosome. Conserved heme-binding motifs and histidines are indicated by blue lines and stars, respectively. Loop regions involved in the *D. soudanensis* WTL bundling interface are highlighted by red boxes. Loop regions that adopt different conformations within *D. soudanensis* WTL bundles are indicated by green lines.

Given the presence of seven distinct states of cytochrome subunits along a single cytochrome fiber, we then investigated their dynamic behavior. Notably, when applying a global helical symmetry during reconstruction, several loop regions appeared smeared and less defined in the resulting map. However, upon relaxing the helical symmetry in the asymmetric reconstruction, these loops became clearly traceable (Fig. 3A and B), suggesting that they are ordered loop structures that adopt different conformations across the seven states. This observation was further validated by calculating the RMSD between the seven subunits, which revealed that these same loop regions undergo moderate conformational changes, rotating out of the subunit to interact with nearby filament (Fig. 3C). Intriguingly, the loops exhibiting conformational changes overlap with the type 1 interface (T128–A131) and are in close proximity to a portion of the type 2 interface (N134–A137) (Fig. 3D).

### *D. soudanensis* bundle interface loops are not conserved among OmcE homologs

**Fig 4:**
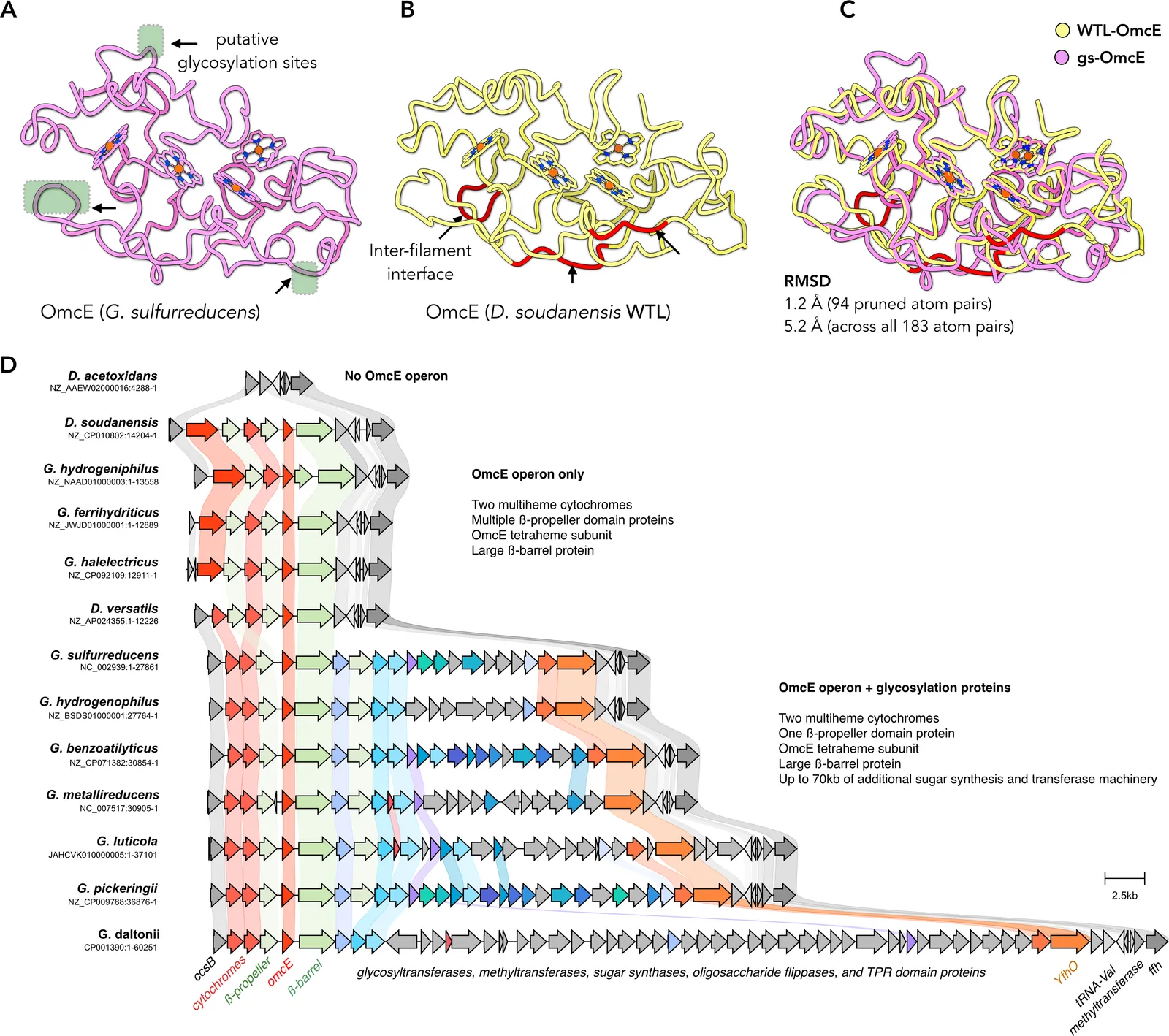
*D. soudanensis* WTL OmcE vs *G. sulfurreducens* OmcE. (**A**) A single OmcE subunit from the cryo-EM structure of the *G. sulfurreducens* OmcE filament. Regions where additional cryo-EM densities were observed are indicated by green squares. (**B**) A single OmcE subunit from the bundled structures of *D. soudanensis* WTL OmcE. Loop regions involved in inter-filament interfaces are colored red. (**C**) Superimposition of the two structures shown in panels **A** and **B**. (**D**) Comparison of the *omcE* gene cluster between *G. sulfurreducens*, *D. soudanensis* WTL, and other bacterial strains.

As the bundling of cytochrome nanowires is a newly observed phenomenon, we were curious whether the amino acids participating in the specific bundling interfaces, or within the loops exhibiting conformational changes, were conserved among other OmcE homologs. Tetraheme cytochrome gene sequences similar to OmcE are widely distributed, so we narrowed our sequence analysis to OmcE-like genes that are potentially capable of assembling into nanowires by including only OmcE homologs for which other genes characteristic of this nanowire were also present, such as genes encoding a large outer membrane β-barrel protein putatively functioning as a secretion pore or anchor, and a β-propeller-domain lipoprotein (see Fig. 4).

Remarkably, putative OmcE homologs were identified across five different phyla, predominantly in Thermodesulfobacteriota and Pseudomonadota, with some examples also found in Aquificota, Campylobacterota, and Nitrospirota. A sequence alignment of homologs from these five phyla revealed several conserved regions, including the four CxxCH motif regions and the other four histidine residues involved in heme coordination. Strikingly, almost none of the *D. soudanensis* loops that facilitate bundle formation were conserved among any subset of these OmcE homologs (Fig. 3D). The only region displaying moderate conservation is N134–A137, which is part of the type 2 interface; however, this limited conservation might be attributed to its relatively close proximity to a conserved histidine residue that coordinates an internal heme.

### Similarities between OmcE nanowire structures

As the *D. soudanensis* nanowire bundle is the second experimentally characterized OmcE nanowire, we investigated its differences from the *G. sulfurreducens* version. The amino acid sequence of *D. soudanensis* OmcE exhibits 41% sequence identity to *G. sulfurreducens* OmcE, and both structures are primarily composed of random coil domains. Similar to *G. sulfurreducens*, *D. soudanensis* OmcE contains ~3% β-strands and ~17% α-helices, with the remaining 80% adopting coil and turn conformations. While the overall architecture of these two cytochromes appears similar, conformational variations are noticeable (Fig. 4A and B). In *G. sulfurreducens* OmcE, extra densities, likely corresponding to glycosylation, were observed (17). In contrast, the cryo-EM map of *D. soudanensis* OmcE did not reveal such densities in non-AI-modified cryo-EM maps, suggesting an absence of post-translational modifications.

Consistent with this observation, the loops containing putative glycosylation sites in *G. sulfurreducens* OmcE are considerably different from those in *D. soudanensis* OmcE. Furthermore, the loops implicated in inter-filament interfaces in the OmcE bundle structure also exhibit distinct conformations compared to their counterparts in *G. sulfurreducens* (Fig. 4A through C). This divergence is reflected in the structural alignment of the two cytochromes (Fig. 4C). The RMSD between 94 pruned atom pairs, encompassing all heme-coordinating regions, is ~1.2 Å, indicating the heme core packing is conserved. However, upon expanding the alignment to include all ~180 atom pairs, the RMSD increases to 5.2 Å, highlighting significant conformational differences in non-heme coordinating regions. These differences are largely attributable to the distinct surface residues, namely those involved in glycosylation versus inter-filament interaction.

As glycosylation appeared to be a major difference between these structures, we compared genomic regions across Desulfuromonadia (Fig. 4D). Nearly all genomes possess a region of shared synteny beginning with purine metabolism (*purHDE*) and heme maturation (*resB*, *ccsB*), and ending with tRNA(Val) modification and signal recognition particle (*ffh*). Some genomes encode an OmcE-containing cluster between these genes. The smallest clusters always encode two mutiheme cytochromes, two β-propeller lipoproteins, the OmcE nanowire subunit, and a large β-barrel protein. This pattern is primarily found in marine, alkaliphilic, and thermophilic genera (classically named *Desulfuromonas*, *Geoalkalibacter*, and *Geothermobacter*). A second, more common pattern contains the same OmcE gene cluster, followed by 15 to 70 genes encoding glycosyltransferases (typically families 2, 4, and 39), sugar modification enzymes, methyltransferases, sugar exporters, tetratricopeptide repeat domain proteins, and transcriptional regulators. These additional glycosylation genes are found primarily in genera associated with freshwater and subsurface habitats, such as *Geobacter* and *Geomonas*. These genomic differences are consistent with our observed lack of glycosylation in *D. soudanensis*.

### Heme arrangements in OmcE-like structures and a possible new OmcE nanowire family

**Fig 5:**
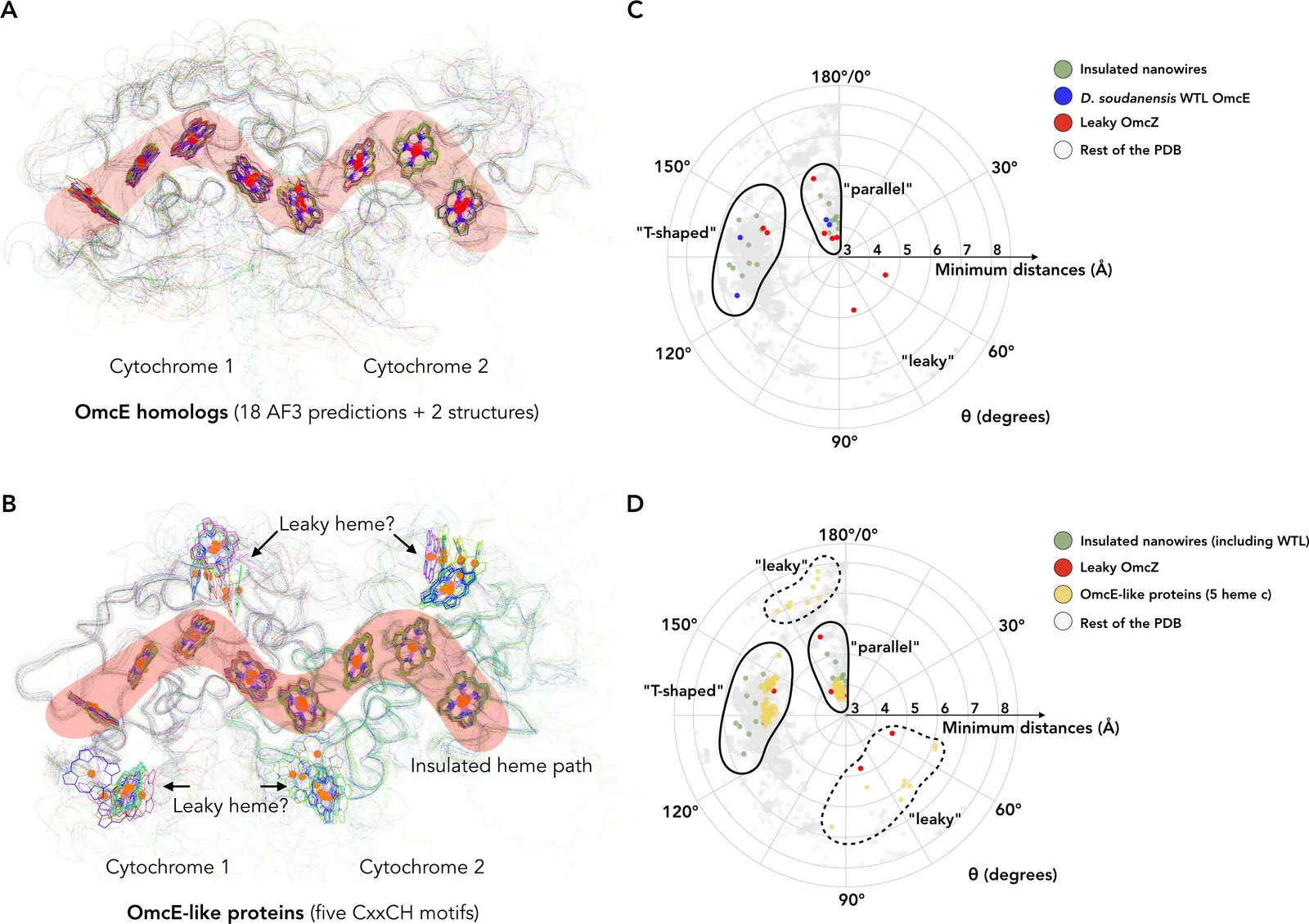
Heme arrangements in OmcE-like proteins. (**A**) Two subunits of OmcE homologs (sequence listed in Fig. 3). Experimentally determined OmcE models were used for *D. soudanensis* WTL and *G. sulfurreducens*. Other OmcE proteins were predicted using AlphaFold3 with heme molecules and aligned to the *D. soudanensis* WTL OmcE model. Heme molecules are shown as sticks, while all protein backbones are displayed with very thin ribbons. (**B**) Similar to panel **A**, but instead of OmcE homologs, this panel shows protein predictions with sequence similarity to OmcE but possessing five CxxCH motifs. These predictions were aligned to the experimentally determined *D. soudanensis* WTL OmcE model. (**C**) Heme-heme orientation plot. One heme can be aligned to an adjacent heme by a rotation and a translation. Only the 25 non-hydrogen atoms in the porphyrin ring were used in the alignment. Heme pairs in all PDB structures were analyzed. Minimum distances refer to the shortest distance between two porphyrin rings, regardless of atom type. The angle θ was determined from the alignment rotation matrix between heme pairs. All porphyrin ring pairs with a minimum distance less than or equal to 8 Å are shown. Porphyrin ring pairs in insulated nanowires, the *D. soudanensis* WTL OmcE, and the “leaky” OmcZ are shown in green, blue, and red, respectively. (**D**) Heme-heme orientation plot similar to panel **C**. Porphyrin ring pairs in insulated nanowires (including *D. soudanensis* WTL OmcE), “leaky” OmcZ, and OmcE-like proteins from panel **B** are shown in green, red, and yellow, respectively.

All known “insulated” cytochrome nanowires, including OmcE (17) and OmcS (19) from *G. sulfurreducens*, pcECN from *P. calidifontis*, and avECN (26) from *A. veneficus*, share a similar arrangement of densely packed hemes. Within this arrangement, the porphyrin rings of heme-heme pairs are in either “parallel” or “T-shaped” alternating configurations. Despite their significant structural and sequence differences, the heme arrangement in the *D. soudanensis* OmcE filament is nearly identical to that of *G. sulfurreducens* (Fig. 5A and B).

Furthermore, AlphaFold predictions of all other OmcE homologs suggest that they also share the same heme arrangement (Fig. 5A). A meta-analysis of heme pairs in all multiheme c-type cytochrome structures deposited in the Protein Data Bank reveals that the vast majority of these pairs are arranged in parallel or T-shaped configurations (26). When we added *D. soudanensis* OmcE to this analysis (Fig. 5B), it clearly fell within these two preferred regions.

The broadly conserved geometry of these heme pairs suggests that a convergent design principle has evolved to allow electron transfer through multiheme c-type cytochromes. It is worth noting that all measured rotation angles for the “parallel” heme pair configuration are between 170 and 180 degrees because the vinyl groups of these heme pairs are positioned on opposite sides of their porphyrin ring planes, resulting in a rotation angle close to 180 degrees, rather than 0 degrees, when the two heme molecules are aligned.

Interestingly, when we searched for OmcE-like proteins via PSI-BLAST, we identified many proteins with additional CxxCH binding motifs, despite maintaining a similar protein length. Most of these proteins belong to uncultured bacterial species distributed across diverse phyla. We were curious about the potential location of these extra hemes, especially if these proteins were to assemble into a nanowire. Therefore, we aligned AlphaFold predictions of these proteins to subunits of the *D. soudanensis* OmcE model (Fig. 5C). Remarkably, these 5-heme OmcE-like proteins still exhibit the conserved insulated heme arrangement, while the additional heme is predicted to be much more solvent-accessible. The extra heme also appears to have a preferable packing location (Fig. 5C). This observation is reminiscent of the “leaky” nanowire OmcZ (18) from *G. sulfurreducens*, where each cytochrome subunit has a solvent-accessible heme. We then plotted the heme-heme pairs of these OmcE-like proteins on the polar plot, revealing two additional clusters originating from the extra heme. One cluster closely resembled that of OmcZ, while the other cluster displayed “stacked” pairs but with greater porphyrin–porphyrin distances (Fig. 5D). This suggests the existence of an uncharacterized nanowire family with properties blending both OmcE and OmcZ.

### Charge transport and redox properties of *D. soudanensis* OmcE bundles

**Fig 6:**
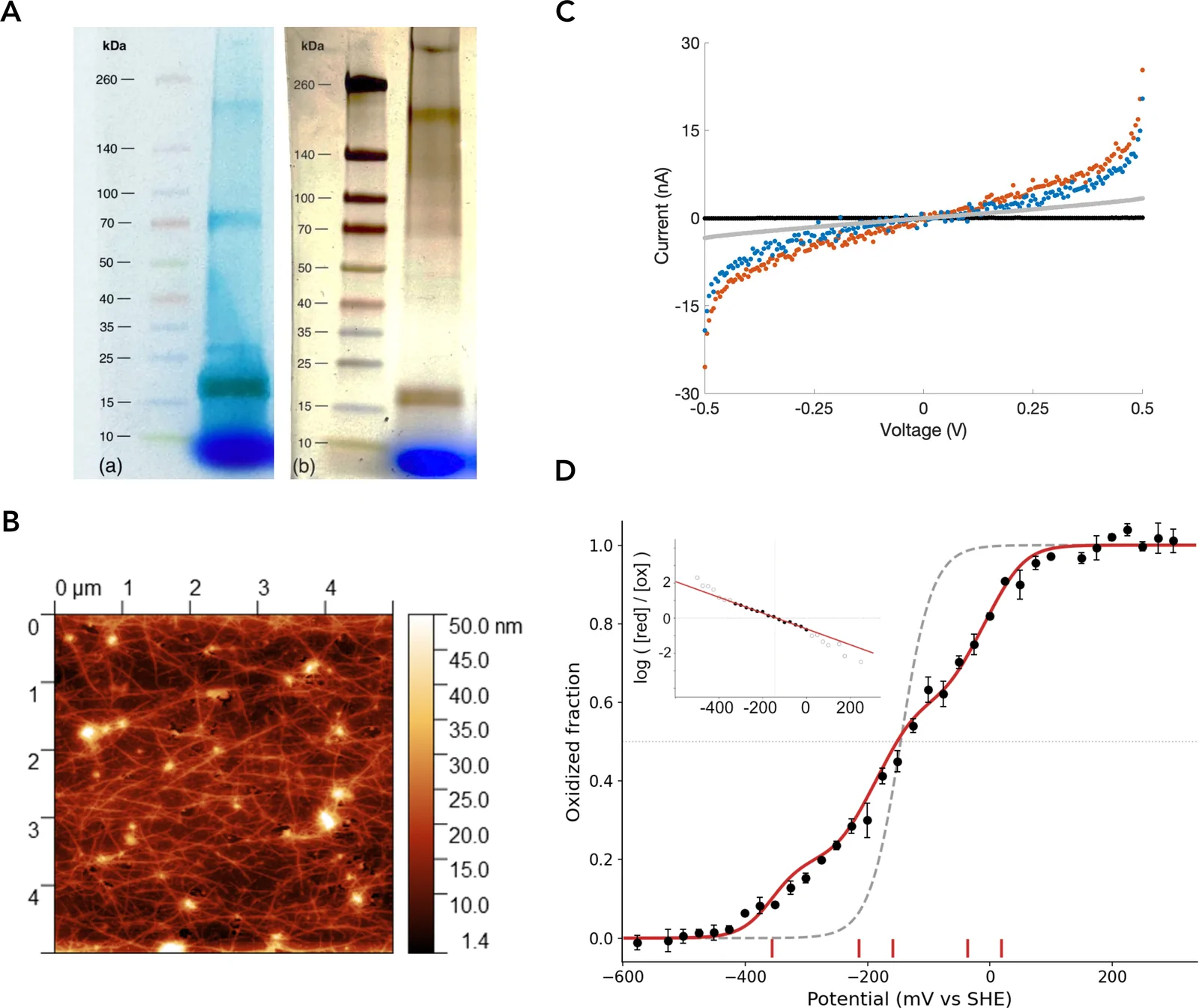
Charge transport and redox characterization of WTL fiber films. (**A**) Purified WTL nanowires analyzed by SDS-PAGE followed by heme staining (left) and silver staining (right). Molecular weight ladder markers (kDa) are shown to the left of the sample lanes. (**B**) Atomic force microscopy topography image of WTL nanowires deposited on mica at 25% concentration diluted in ultrapure water. (**C**) Two-point current–voltage (I–V) characteristics of WTL fiber films deposited onto interdigitated electrodes (IDEs). The black points are from a blank IDE with no sample deposited, the gray points are a buffer deposition control without fibers, the blue points are the prepared WTL solution diluted with ethanolamine to 1% of the initial concentration, and the orange points are the prepared WTL solution diluted with ethanolamine to 5% of the initial concentration. (**D**) Redox titration to determine midpoint potential and heme electrostatic coupling in *D. soudanensis* OmcE bundles. Oxidized fraction (mean ± SD) from the α-band (550–555 nm) vs applied potential, with a single-site (*n* = 1) Nernst fit (gray dashed line). The apparent midpoint potential is −145 ± 1 mV vs SHE. The best-fit sum of five one-electron components (red solid line) reproduces the full breadth of the redox transition. Red ticks indicate the five apparent midpoint potentials from the best-fit model (−356, −214, −159, −37, and +18 mV vs SHE). Top left is the Hill analysis of *D. soudanensis* OmcE bundle redox titration. The slope of log_10_([reduced]/[oxidized]) through the midpoint region (0.12 < *f*_ox_ < 0.88) gives a Hill coefficient *n*_*H*_ = 0.27, well below the value of 1 expected for a single non-interacting heme, indicating a broadened, heterogeneous multiheme transition.

To confirm the electronic functionality of these OmcE assemblies, we performed biochemical and electrical characterization on enriched WTL fibers (Fig. 6). Denaturing gel electrophoresis and heme-staining confirmed the presence of a single major cytochrome species (Fig. 6A), while atomic force microscopy (AFM) verified that the bundle morphology was preserved in purified preparations (Fig. 6B). Despite the high degree of similarity in heme arrangements between various “insulated” nanowires, the conductivity of OmcE-class filaments has never been independently established. Therefore, we measured the current–voltage (I–V) characteristics of dried WTL fiber films deposited onto interdigitated electrodes (IDE) via drop-casting (Fig. 6C). The resulting films exhibited concentration-dependent conductance across a voltage sweep from −0.5 V to +0.5 V, with the 5% and 1% dilutions showing significantly greater conductance than buffer-only controls.

While these film-level measurements provide a necessary proof of principle that bulk preparations containing these bundled assemblies are conductive, we acknowledge that they represent an ensemble measurement of dried material. It has been discussed previously that reliable technology does not yet exist for measuring the conductivity of individual, fully hydrated filaments of a known and confirmed composition (26). The development of such high-precision instrumentation remains a critical goal for the field of electromicrobiology to bridge the gap between atomic structures and *in vivo* electronic flux. Therefore, we have established a baseline conductivity for bulk films containing these novel assemblies, acknowledging that isolating the specific electronic contribution of the anti-parallel bundle interface will require direct measurements on individual, fully hydrated bundles or single filaments as more advanced measurement technologies become available.

To better understand electron transfer processes in *D. soudanensis* OmcE bundles, we characterized their solution redox properties. The heme content of the purified *D. soudanensis* OmcE stock was first quantified by the pyridine hemochromogen assay to enable accurate sample preparation for spectroelectrochemistry (Fig. S6A). The reduced-minus-oxidized difference spectrum displayed the characteristic ferrous α- and β-bands at 550 and 521 nm, consistent with covalently bound *c*-type heme (Fig. S6B). Using the difference absorbance at 550 and 535 nm (Δ*A* = 0.336 ± .008) and the pyridine hemochrome reduced/oxidized spectra (Fig. S6C), the stock heme concentration was calculated as 28.1 ± 0.6 µM or about 7 µM protein, assuming four hemes per OmcE subunit as indicated by the cryo-EM structure. The α-band extinction coefficient of the reduced nanowires at 552 nm was ε = 31 mM^−1^cm^−1^ per heme, within the range reported for other bis-histidine multiheme c-type cytochromes (STC, 23 mM^−1^ cm^−1^ at 552 nm [49]; cyt c_550_, 35 mM^−1^ cm^−1^ at 550 nm [50]; cyt c, 29 mM^−1^ cm^−1^ at 550 nm [51]).

Spectroelectrochemical titration of the OmcE bundles produced characteristic redox-dependent spectral changes in the Soret band and α- and β-band absorbance peaks at 552 nm and ~523 nm (Fig. S7). The normalized α-band absorbance plotted vs applied potential gave a sigmoidal redox titration curve (Fig. 6D). A single-site Nernst fit indicated an apparent midpoint potential of *E*_*m*_ = −145 ± 1 mV versus SHE, although the redox-active window is much broader than that described by this model. The Hill coefficient of the titration was *n*_*H*_ = 0.27, far below the value of unity expected for a single non-interacting center, suggesting the involvement of multiple electrostatically coupled hemes or multiple independent hemes with distinct midpoint potentials in the redox behavior (Fig. 6D) (52, 53).

The titration data were fit to sums of *n* one-electron Nernst components by choosing the number of components with the information criterion, *F*-test, and bootstrap procedures described in Materials and Methods. The best fit resolved five Nernstian components that together reproduce the full breadth of the transition (Fig. 6D), and all three information criteria reach their best (lowest) values with five components (Fig. S8). The five apparent midpoint potentials (−356, −214, −159, −37, and +18 mV vs SHE) span a ~370 mV window, but these values are parameterization-dependent and individually weakly constrained. The component count and the width of the redox-active window, on the other hand, are robust outputs of the analysis. These results represent the first redox characterization of an OmcE-family cytochrome nanowire.

The midpoint potential and potential range of *D. soudanensis* OmcE bundles are comparable to other extracellular electron transfer multiheme *c-*type cytochromes. Hemes in *Geobacter* OmcS nanowires titrate over a ~360 mV window with an apparent midpoint potential of −130 mV vs SHE (52). The redox window of the octaheme, nanowire-forming *Geobacter* OmcZ also spans a range of ~360 mV, with eight non-equivalent hemes, but has a substantially lower apparent midpoint potential of −220 mV vs SHE (54). The periplasmic *Geobacter* triheme cytochromes PpcA–E, on the other hand, exhibit potential windows of ~265 to 285 mV, but they have similar apparent midpoint potentials (−117 to −143 mV vs SHE) to *D. soudanensis* OmcE and *Geobacter* OmcS (55, 56). The similar redox windows of cytochrome nanowires could indicate electrostatic coupling across the same number of hemes (~5) within the nanowires, despite differences in the number of hemes per subunit (4, 6, and 8, respectively). In OmcE nanowires (four hemes per subunit), this coupling would extend across subunit interfaces such that the reduction or oxidation of a heme in one subunit could influence the redox properties of a heme in an adjacent subunit. These comparisons suggest that electrostatic coupling between hemes may be a stronger determining factor of cytochrome nanowire redox and charge transport properties—and a more relevant experimental characteristic—than their theoretical redox potentials in isolation. This conclusion is also consistent with the narrower potential window of the PpcA–E cytochromes (three hemes per subunit), which do not form fiber or extended assemblies and therefore restrict heme coupling to those within each protein.

### Bundling of OmcE cytochrome nanowires: a common phenomenon?

The bundling effect observed in *D. soudanensis* OmcE is not unique. We also observed bundle formation in extracellular appendages sheared from *Geobacter metallireducens* (Fig. S9), an iron-reducing bacterium (57) often studied as a partner in direct interspecies electron transfer (58). *G. metallireducens* is a freshwater isolate phylogenetically closer to *G. sulfurreducens* than to *D. soudanensis*. Notably, *G. metallireducens* does not encode OmcS, but it does encode OmcE and OmcZ. Further, the gene cluster in *G. metallireducens* is highly similar to *G. sulfurreducens* and encodes a clear glycosylation cluster.

Using cryo-EM to analyze the *G. metallireducens* extracellular appendages, we identified type IV pili made of PilA-N and PilA-C and putative cytochrome nanowires. These cytochrome nanowires were present both as single filaments and in large bundles. 2D class averages of the cytochrome bundles showed specific interfaces between the filaments, resolving multiple filaments in one average (Fig. S9). The average power spectrum revealed a helical symmetry for a single cytochrome nanowire of 29 Å rise and 60° twist, consistent with known symmetries for OmcE nanowires. Furthermore, the protein subunits within these 2D classes displayed the “Pac-Man” shape, consistent with other experimentally determined OmcE structures (Fig. S9). While the limited number of orientations resulted in only six views, preventing reconstruction of the bundle to near-atomic resolution, our observations confirm that bundling of OmcE nanowires occurs in *G. metallireducens* as well. If this quaternary arrangement is more common than previously recognized, future cryo-EM studies should take this into consideration and examine these more difficult-to-image bundles as well as individual filaments.

## DISCUSSION

Recent advances in cryo-EM (59) and deep-learning methods (60, 61) have significantly reshaped the field of microbial electron transfer, and current techniques have proven very valuable for *de novo* identification of extracellular protein polymers without prior knowledge (62–64). In this study, we used cryo-EM to examine the sheared total appendages from the iron-reducing bacterium *D. soudanensis* WTL (45). Strikingly, the only type of filament observed formed thick bundles of anti-parallel packed multiheme c-type cytochromes, based on a subunit homologous to *G. sulfurreducens* OmcE. This is the first functionally relevant quaternary state reported for a nanowire structure.

This is also the first instance of a bacterial cytochrome nanowire structure being experimentally determined outside of *G. sulfurreducens*. Furthermore, we showed that another organism, *G. metallireducens,* also produces cytochrome filaments in bundles consistent with the same OmcE subunit, indicating that such bundling is not a unique case. The unexpected observation of bundling in cytochrome nanowires suggests possible roles in colonization and biofilm formation and reveals that cytochrome nanowire structures can be more complex than initially anticipated. It also provides insights into how such architecture might facilitate electron transfer between cells.

As previously reported (45), *D. soudanensis* WTL grows more slowly than many better-known metal-reducing bacteria, with a doubling time of approximately 13 h. For an organism isolated from a 10°C low-nutrient metal-rich brine, where significant energy is likely devoted simply to maintenance and ion homeostasis (65, 66), the large quantity of extracellular cytochrome bundles represents a substantial energy expenditure. Heme-rich *c*-type cytochromes are energetically expensive to synthesize (67), as a filament could contain over 10,000 protein subunits and 40,000 heme cofactors, and each bacterium appears to produce at minimum several dozen such nanowires. Why *D. soudanensis* invests a large amount of energy in this single part of its respiratory chain remains an open question.

How cytochrome nanowires are secreted and assembled on the cell surface also remains unanswered. Even among bundling pili or secreted protein bundles that share a similar “jelly-roll” fold (*P. calidifontis* archaeal bundling pili, *B. subtilis* TasA, and *P. aeruginosa* CupE), the secretion systems likely differ. The secretion mechanisms for the first two remain unclear, whereas *P. aeruginosa* CupE clearly utilizes a chaperone–usher pathway (68–70), with the pilus mounted on an outer membrane β-barrel usher. In *G. sulfurreducens*, the insulated cytochrome nanowire OmcS was initially proposed to be secreted via the ATP-dependent type IV pilus, analogous to the type II secretion system (71). However, a more recent hypothesis suggests that it is instead mounted on the outer membrane using a porin-like pathway aided by several other proteins (72). Considering the large number of cytochrome nanowires produced by each cell, a porin model appears more energetically efficient and is consistent with the presence of large β-barrel proteins in most OmcE and OmcS genomic regions.

These questions of secretion illustrate how cryo-EM nanowire structures resolve some questions regarding electron transfer while simultaneously raising many more involving physiology. For example, if OmcE assembly occurs via simple polymerization analogous to chaperone–usher systems, how is it terminated? Or does it even need to be terminated? In some classic CUP pili assembly systems, the process is halted by the incorporation of a slightly different “anchor” pilin. This prevents the chaperone from dissociating, thereby blocking the addition of further major pilins (73). An “anchor” cytochrome with a similar function might operate in *D. soudanensis*. Because the interface between OmcE subunits that form the filament features an unsatisfied heme requiring an additional histidine from the next subunit, an ideal anchor protein would possess nine histidines and four hemes, with a structure resembling OmcE. Using AlphaFold3, we identified one protein, Dsoud_2415, that meets these criteria in *D. soudanensis* WTL. However, this protein is not located within the same gene cluster as OmcE, while an anchor pilin typically co-evolves with its major filament subunit, as observed in CUP pili (74, 75), making this hypothesis a bit less likely. Similar to the problem of termination is one of initiation: what is the structure of the filament’s tip? Could it be a protein similar to OmcE but with more solvent-exposed hemes to enhance contact with minerals? Currently, no such candidate appears encoded in the OmcE operons.

**Fig 7:**
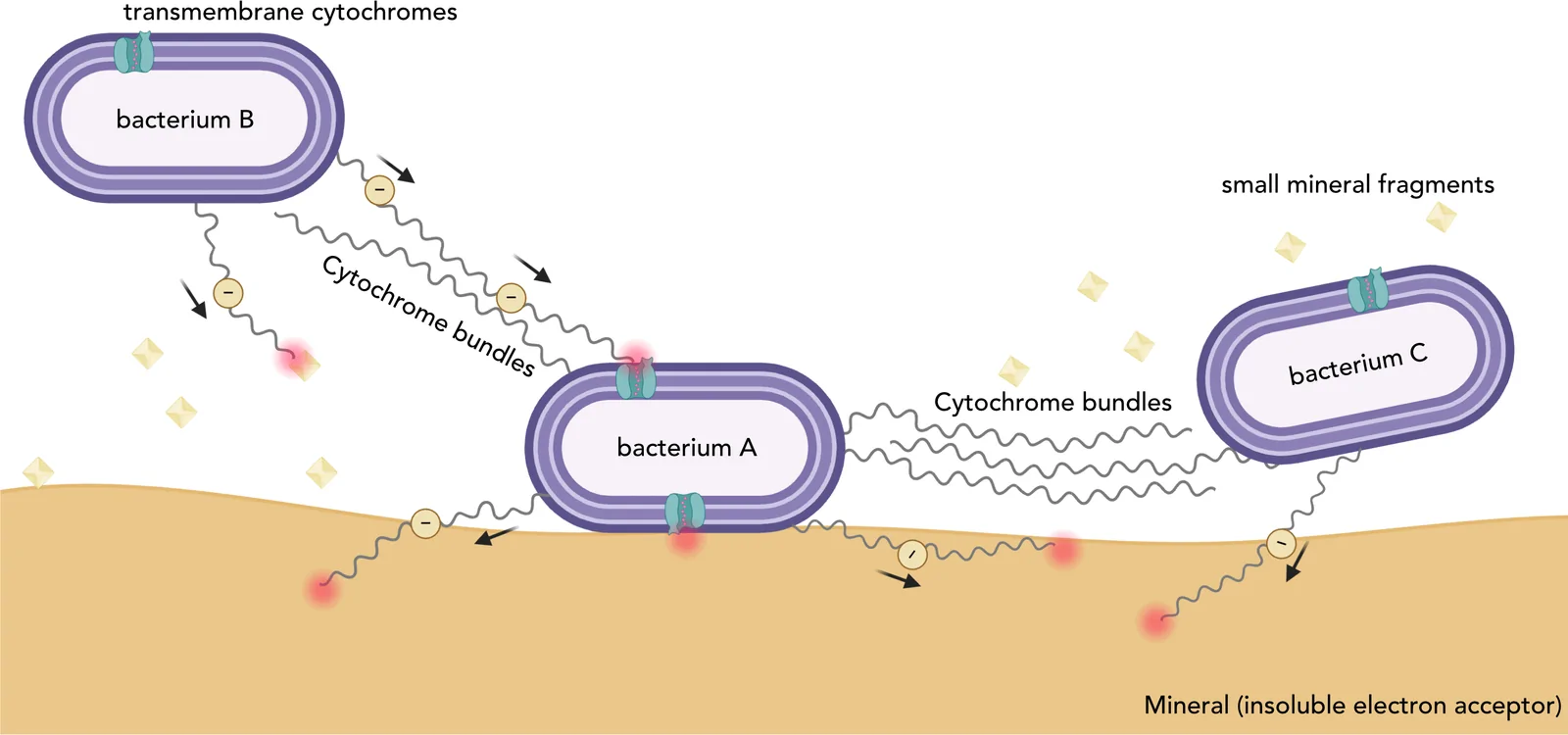
Schematic of potential cytochrome bundle functions within *D. soudanensis* WTL biofilms. Three representative bacterial cells (labeled bacterium A–C) are depicted. OmcE nanowires are represented by thin, curved lines, and simplified bundles are indicated. Putative transmembrane or membrane-embedded cytochromes are represented by green cylinders. Minerals and mineral fragments are shown in yellow. Potential electron transfer events are indicated by a red halo effect.

Ultimately, what could be the advantage of a cell producing bundles rather than individual cytochrome filaments? A simple explanation is that bundling could protect filaments from degradation or phage recognition or protect them against mechanical shearing. However, given the invariant anti-parallel structure, an evolutionary force driving bundling for cell-cell interaction seems compelling (Fig. 7). If a portion of the biofilm is in direct contact with minerals, daughter cells are more likely to remain linked simply because the bundled nanowires keep cells close. A more intriguing possibility involves potential electron transfer between cells. The heme-to-heme distance between the heme cores inside each filament is at least 25 Å, which is too great for efficient electron transfer between adjacent nanowires in the bundles. However, cell-to-cell electron transfer would be possible if the end of each nanowire makes contact with a neighboring cell. This interaction would create a highly insulated pathway for one cell to transfer electrons to a distant acceptor, using another cell as a transfer station (Fig. 7).

In summary, our study has identified a new higher-order structure for the OmcE cytochrome nanowire, based on anti-parallel bundles found in both *D. soudanensis* WTL and *G. metallireducens*. The near-atomic-resolution structure of *D. soudanensis* WTL bundles suggests that these nanowires could serve a dual function in both electron transfer and biofilm formation, implying a more complex role in anaerobic respiration than previously understood. This work suggests that cytochrome nanowires are a widespread tool used by bacteria beyond *G. sulfurreducens* and highlights the need for future research to elucidate the mechanisms of cytochrome nanowire secretion, assembly, and function.

## MATERIALS AND METHODS

### *D. soudanensis* WTL cytochrome nanowire preparation

*D. soudanensis* cells were collected from 1 L of culture in a modified Soudan Mine (SM) medium. The medium was degassed in 80:20 N_2_:CO_2_ and contained 20 mM sodium acetate, 40 mM sodium fumarate, 10 ml/L mineral mixture (23), 0.04 mM magnesium sulfate, 19 mM ammonium chloride, 0.37 mM potassium phosphate monobasic, 75 mM calcium chloride, 37 mM magnesium chloride, and 135 mM sodium chloride. The pH was adjusted to 6.8 before adding 21.5 mM sodium bicarbonate (45). When the density reached 0.6 of OD_600_, cell culture was harvested by centrifugation for 20 min at 8,000 × *g*. Cytochrome filament abundance and bundle formation were examined by negative staining. Extracellular filaments were sheared by drawing and expelling the cell suspension through a 5 mL syringe attached to a 25 G × 7/8-inch needle 10 times. The debris was subsequently removed by centrifugation for 20 min at 8,000 × *g*. The sheared filaments were then resuspended in 150 mM ethanolamine buffer (pH 10.5) before cryo-EM imaging.

### *Geobacter metallireducens* cytochrome nanowire preparation

*G. metallireducens* biofilms were grown on 9 cm^2^ graphite electrodes poised at 0 V vs SHE in 100 mL bioreactors in mineral medium with only 40 mM sodium acetate, without sodium nitrate, Fe citrate, or sodium tungstate (23). To increase the biomass of the biofilm, bioreactors were supplemented with five successive rounds of 40 mM acetate supplementation when electrode current decreased to near 0 amps. Biofilm was scraped off the graphite and resuspended in 50 mM Tris buffer at pH 7.5 with 0.25 U/mL benzonase. The filaments were sheared off by drawing and expelling cell suspension through a 5 mL syringe attached to a 25 G × 7/8-inch needle 10 times. The debris was removed by centrifugation for 20 min at 8,000 × *g*.

### Negative staining electron microscopy

For negative staining imaging, continuous carbon grids (EMS, CF300-Cu) were glow-discharged for 30 s at 30 mA, with the carbon side facing up. Subsequently, 3.5 μL medium culture containing *D. soudanensis* WTL cells was directly applied to the carbon side of the glow-discharged grid and allowed to incubate for 20 s. The grid was then back-blotted by placing the copper side on filter paper (Whatman) and gently dragging it downward against the filter paper to remove the excess sample. Immediately following this, 10 μL of ultrapure water was dropped onto the grid surface and quickly back-blotted as before; this washing step was repeated once. Following the second wash, 8 μL of 2% uranyl acetate (EMS) was placed onto the grid and back-blotted using the same procedure. The grid was then allowed to air dry for 1 min. TEM images were collected on a 200 keV JEOL JEM-1400 transmission electron microscope equipped with an AMT NanoSprint43L MarkII camera at the University of Alabama at Birmingham’s High Resolution Imaging Facility.

### Cryo-EM conditions and image processing

For cryo-EM imaging, the enriched cytochrome nanowire sample (4.5 μL) was applied to glow-discharged lacey carbon grids and then plunge-frozen using an EM GP2 Plunge Freezer (Leica). Cryo-EM micrographs were collected on a 300 keV Titan Krios microscope equipped with a K3 camera at a pixel size of 1.07 Å and a total dose of 50 e⁻/Å². Preliminary cryo-EM analysis using a non-tilted data set indicated only seven distinct views of cytochrome subunits within the cytochrome bundles, suggesting a preferred orientation, a rare problem for filaments. This is largely because the helical twist is 51.4°, almost exactly 360° divided by 7, and the OmcE bundles primarily form a flat, single-layer network. Consequently, all single cytochrome subunits within individual filaments are restricted to one of those seven specific views. To overcome this problem, we performed additional tilted data collections at +12° and −12° for grids with the thinnest ice and collected an additional data set on a thicker ice grid using +4° tilted data collection to capture all possible azimuthal views of the cytochrome subunits (Fig. S3).

The final cryo-EM workflow began with patch motion correction and CTF estimation in cryoSPARC (76–78). Subsequently, automated particle segment picking was performed using the “Filament Tracer” function with a 30-pixel shift between adjacent boxes. All auto-picked particles underwent multiple rounds of 2D classification, and particles belonging to poor 2D class averages were removed. High-quality particles were selected based on whether the class averages clearly resolved cytochrome building blocks across multiple filaments, indicating that the inter-filament interfaces were specific and could be successfully averaged between particles. Next, we aimed to first obtain a helical reconstruction of a single filament within the bundles. Potential helical symmetries were calculated from averaged power spectra generated from the raw particles (512-pixel box). Only one possibility was evident, given the clear meridian layer line and another layer line near the equator (79). Next, 3D reconstruction was performed using “Helical Refinement,” followed by “Local CTF refinement,” and another round of “Helical Refinement” using the CTF-refined particles. The resolution of the helical reconstruction was estimated to be approximately 3.4 Å using map-to-map FSC.

To resolve the higher-order bundle structure, the refined particles and the 3.4 Å helical volume of the single filament were used as a high-resolution “seed” to initialize 3D classification without applied symmetry. Additional filaments making contact with the central filament used as a reference began to emerge. Four out of 10 classes exhibited at least one clear additional filament, and visual inspection of these volumes using ChimeraX (80) indicated that all had the same inter-filament interfaces but differed in the orientations of their central filaments. These four classes were subsequently merged for an asymmetric reconstruction pipeline that included Homogeneous Refinement, Local Refinement, Local CTF Refinement, and a final round of Local Refinement. Through this approach, the final reconstruction reached a global resolution of 3.1 Å, as estimated by map-to-map FSC. During the modeling stage (see below), we observed slight structural variations between subunits. This is consistent with the observation that relaxing helical symmetry yielded a slightly higher resolution asymmetric reconstruction. Maps used for the model-to-map resolution estimation were sharpened using B-factor-based “Local Filtering” within cryoSPARC. For better visualization, the unsharpened asymmetric map was modified using the deep-learning-based algorithm DeepEMHancer (81). The relevant statistics, using a non-AI sharpened map only, are listed in Table S1.

### Model building of cytochrome bundles

Model building began with identifying the correct cytochrome building block from the experimental cryo-EM map. From the helical reconstruction map, we used ModelAngelo (60) to successfully trace most of the protein backbone, and the predicted protein sequence was used to perform a BLAST search against the *D. soudanensis* WTL proteome. An OmcE homolog, UniProt ID A0A0M4DKA8 (ORF name: DSOUD_2954; gene name: *omcE*), was identified as the best hit, having ~50% sequence identity to the ModelAngelo-predicted sequence. To validate this, full-length modeling was performed for DSOUD_2954 and all other OmcE-like proteins within the proteome, and DSOUD_2954 was the only protein that could fully account for the cryo-EM map (Fig. S10).

For the modeling process, the AlphaFold3-predicted model of a single *D. soudanensis* WTL OmcE cytochrome subunit initially exhibited poor geometry, with a Ramachandran favored percentage below 60%. To address this, we employed RosettaCM (82) rebuilding scripts with heme ligands, generating over 2,000 trajectory models. A chimera model for subsequent refinement was constructed from these models by selecting regions with top geometry statistics and manually choosing those that best matched the cryo-EM map based on visual inspection. Furthermore, accurately placing heme molecules into the cryo-EM map at this resolution without prior knowledge is challenging because existing software lacks geometry restraints for amino acid–heme interactions. Therefore, to improve the refinement of heme-interacting regions at this resolution, bond and angle restraints for the heme molecule itself, His-Fe bonds, and Cys-heme thioether bonds were implemented based on geometries observed in high-resolution crystal structures such as NrfB (83) (PDB ID: 2P0B) and NrfHA (84) (PDB ID: 2J7A). Similar refinement strategies have been employed for previous cytochrome nanowire studies (17, 18, 26). Individual cytochrome subunits were manually adjusted against the asymmetric reconstruction map in Coot (85), and the resulting bundle model was then real-space refined (86) against the asymmetric map. Model quality was assessed using MolProbity (87). Refinement statistics for the WTL bundles are presented in Table S1.

### Structural analysis of heme molecules in heme-containing PDB structures

All experimental structural coordinates with heme ligands were downloaded from the Protein Data Bank (PDB). Structural predictions with heme molecules were generated using the AlphaFold3 (44) online server (https://alphafoldserver.com/). All possible heme pairs were then filtered, retaining only those with a minimum distance less than or equal to 8 Å between the two porphyrin rings, using the “contact” command in UCSF ChimeraX (80). For each qualifying pair, the rotation matrix between the two hemes was generated in ChimeraX using the “align” command. The rotation angle, θ, was either calculated from the rotation matrix or obtained directly from the ChimeraX command reply log.

### I–V conductivity measurements

WTL nanowires were prepared using the same approach as for cryo-EM data collection, with an additional enrichment step for film formation. *D. soudanensis* OmcE nanowires were precipitated by adding ammonium sulfate to the clarified supernatant to a final concentration of 12.5% (wt/vol). The solution was incubated overnight at 4°C and then centrifuged at 13,000 × *g* for 1 h. The resulting red pellet was resuspended in 500 µL of 150 mM ethanolamine buffer (pH 10.5). Nanowire enrichment was confirmed by SDS-PAGE followed by silver and heme staining (Fig. 6).

Interdigitated electrodes (IDEs) were fabricated at the Nano3 facility (UCSD). Each IDE substrate consisted of 100 parallel gold lines (60 nm Au on a 5 nm Ti adhesion layer) on Pyrex, with 5 μm line widths and 5 μm spacing. Before use, IDEs were cleaned by sonication in acetone, rinsed with isopropanol and ultrapure water, and dried under filtered compressed air. Immediately prior to sample deposition, IDEs were cleaned with oxygen plasma (100W, 10 min) to ensure a hydrophilic surface. WTL nanowire films were prepared by drop-casting 4 μL of the nanowire solution onto the active area of the IDE. After a 5-minute incubation, excess solution was wicked away with filter paper. To remove residual buffer salts, the film was rinsed three times by depositing 4 μL of ultrapure water and immediately wicking it away, ensuring that the film did not fully dry between rinses. The samples were then dried in a vacuum desiccator for 1 h. Control samples were prepared identically using 150 mM ethanolamine buffer (pH 10.5) without nanowires. Electronic measurements were performed using a two-point probe configuration on a Signatone S-725-SRM probe station. Spring-loaded probe tips contacted the gold pads, and data were collected using a Keithley 2612B System SourceMeter. For each sample, the current was recorded during 10 consecutive voltage sweeps from −0.5 V to +0.5 V. The current values at each voltage step were averaged across the 10 sweeps to generate the final I–V profiles.

### Atomic force microscopy of WTL bundles

To further validate the morphology of the purified filaments, 4 μL of WTL nanowire solution (diluted to 25% in ultrapure water) was drop-cast onto a freshly cleaved V-5 grade mica surface over a 0.25 cm^2^ area. Following a 5-minute incubation, excess solution was wicked away with filter paper. To remove residual buffer salts, the sample was rinsed three times with 4 μL of ultrapure water, ensuring that the film remained hydrated between rinses, and was subsequently dried in a vacuum desiccator for 1 h. Topography images were acquired using a Bruker Dimension ICON AFM operating in ScanAsyst-Air mode. Measurements were performed using ScanAsyst-Air probes (nominal spring constant of 0.4 N m^−1^), with the set point and gain settings managed by the ScanAsyst auto-control software.

### Spectroelectrochemistry of WTL bundles

Spectroelectrochemical redox titrations of purified *Desulfuromonas soudanensis* WTL OmcE nanowire bundles were performed in a Honeycomb Spectroelectrochemical Cell (Pine Research Instrumentation, Durham, NC) fitted with a gold honeycomb screen-printed electrode as the working electrode, the integrated gold counter electrode on the same electrode card, and a silver-wire (Ag^+^/Ag^0^) quasi-reference electrode.

Purified WTL OmcE bundles at 28.1 µM heme were prepared in 150 mM ethanolamine (Sigma-Aldrich, E9508, ≥ 98%), adjusted to pH 10.5 with 10 N NaOH (Fisher Chemical, E584, reagent grade). To mediate electron transfer between the electrode and the protein, a cocktail of 19 redox mediators—methyl viologen (Thermo Scientific, 227320010, 98%), benzyl viologen dichloride (Sigma-Aldrich, 271845, 97%), neutral red (Thermo Scientific, J63054.03), safranine O (Thermo Scientific, B21674.09), sodium anthraquinone-2-sulfonate monohydrate (TCI, A0508, >98.0%), disodium anthraquinone-2,6-disulfonate (AA Blocks), 2-hydroxy-1,4-naphthoquinone (Thermo Scientific, 121630100, 99%), 1,4-naphthoquinone (Sigma-Aldrich, 152757, 97%), 1,2-naphthoquinone (Acros Organics, 171230010, 95%, tech.), juglone (Alfa Aesar, H28343.03, 99%), phenazine (TCI, P0082, >99.0%), phenazine methosulfate (Sigma-Aldrich, P9625), phenazine ethosulfate (Sigma-Aldrich, P4544, ≥95%), methylene blue trihydrate (Fisher Chemical, M291-25, certified biological stain), resorufin (Sigma-Aldrich, 73144, 95%), duroquinone (Sigma-Aldrich, D223204, 97%), indigo carmine (TCI, I0214, >95.0%), potassium indigotrisulfonate (Thermo Scientific, 215170010), and gallocyanine (Thermo Scientific, 154430100)—each at a final concentration of 1 mM—was prepared as a stock solution in a 7:3 (vol/vol) H_2_O: DMSO (Fisher Chemical, D136-1) mixture. The stock was added to the sample to give a final concentration of 10 µM each (a 1:100 dilution; 3 µL stock into 297 µL of protein sample), following established *Geobacter* cytochrome spectroelectrochemistry protocols (88). All measurements were performed inside an anaerobic glovebox under an N_2_ atmosphere with O_2_ maintained below 5 ppm.

The applied potential was stepped from +175 mV to −700 mV versus the Ag wire reference in 25 mV increments using a WaveDriver potentiostat (Pine Research Instrumentation, Durham, NC). At each step, the sample was allowed to equilibrate for 3 min before a full UV-visible absorbance spectrum (350–700 nm) was recorded on an Agilent Cary 60 UV-visible spectrophotometer (Agilent Technologies). Two to four spectra were recorded at each potential within a single titration. Reported values are the mean ± SD across these technical replicates. The Ag wire reference electrode potential in the solution was measured as −72.9 mV vs Ag/AgCl electrode, and potential values are reported vs SHE (saturated KCl Ag/AgCl electrode = +197 vs SHE).

### Heme quantification by pyridine hemochromogen assay

The heme concentration of the WTL OmcE stock was determined by the pyridine hemochromogen method of Berry and Trumpower (89). Protein was diluted twofold into pyridine (EMD, PX2020-6, GR ACS)/NaOH to form the bis-pyridine hemochromogen, and the ferric (oxidized by addition of potassium ferricyanide [J.T. Baker, 3104-01, ACS reagent]) and ferrous (reduced by addition of sodium dithionite [Sigma-Aldrich, 71699, ≥82%]) spectra were recorded over 380–950 nm in a 1 cm path-length quartz cuvette. Heme *c* was quantified from the reduced-minus-oxidized difference absorbance at the 550–535 nm wavelength, using the *c*-type hemochromogen difference extinction coefficient Δε_550–535_ = 23,970 M^−1^ cm^−1^ (89) and correcting for the twofold dilution.

The per-heme molar absorption coefficient of WTL OmcE at the α-band was determined by combining the hemochromogen-derived heme concentration with the α-band absorbance of the native protein. The protein was oxidized with a slight excess of potassium ferricyanide (≈0.3 mM final), and a parallel aliquot was fully reduced with sodium dithionite. Spectra were corrected for Rayleigh–Mie scattering using the same Rayleigh–Mie–Offset (RMO) procedure applied to the spectroelectrochemical data (see spectral processing methods below). The α-band absorption coefficient was calculated as ε_550–535_ = Δ*A*_550–535_/[heme], where Δ*A*_550–535_ is the reduced-minus-oxidized absorbance difference between the α-band peak (550 nm) and the reference wavelength (535 nm) and [heme] is the heme concentration determined by the hemochromogen assay. The same 550–535 nm absorbance difference was used for both the heme quantification and the extinction-coefficient determination, such that both are referenced to the same difference coefficient, Δε_550–535_ = 23,970 M⁻¹cm⁻¹ (89), used throughout. Because the α-band lies in a spectral region where scattering is small, the coefficient was insensitive to the scattering correction (<3% change).

### Spectral processing and determination of the midpoint potential

All data processing was performed in Python (NumPy and SciPy) (90). Because the WTL OmcE bundles are large (~150 nm diameter) light-scattering assemblies, raw spectra carried a wavelength-dependent baseline arising from Rayleigh–Mie scattering. Each spectrum was corrected using the Rayleigh–Mie–Offset (RMO) method of Basalla and Kendrick (91), in which the chromophore-free region (600–700 nm) is fit to OD(λ) =*k* λ^−*n*^ + *b,* where *k* is the scattering amplitude, *b* is the wavelength-independent turbidity/instrument offset, and *n* is the scattering exponent. For particles in this size regime, scattering falls between the Rayleigh (λ^−4^) and large-particle Mie (λ^−1^) limits (91). *n* was therefore fixed at 2, and *k* and *b* were determined by least-squares fitting over 600–700 nm for each spectrum independently. The resulting baseline was subtracted across the full wavelength range. Corrected spectra were then smoothed with a Savitzky-Golay filter (92) (11-point window, third-order polynomial), which suppresses high-frequency noise while preserving peak position and shape.

The redox state of the hemes was tracked through the reduced α-band, measured as the mean absorbance over 550–555 nm (peak maximum at 552 nm). For each applied potential, the oxidized fraction *f*_ox_ was computed as *f*_ox_(*E*) = [*A*(*E*) − *A*_red_]/[*A*_ox_ − *A*_red_], where *A*(*E*) is the α-band absorbance at potential *E* and *A*_ox_ and *A*_red_ are reference absorbances defined as the mean of the four most-oxidized spectra (at +175, +150, and +125 mV vs Ag wire) and the four most reduced spectra (at −700, −650, −625, and −600 mV vs Ag wire) of the native protein, respectively.

The midpoint potential was obtained by fitting *f*_ox_ versus *E* to a single-site (one-electron) Nernst equation (52), *f*_ox_(*E*) = 1/{1 + exp[−(*F*/*RT*)(*E − E*_m_)]}, where *F* is the Faraday constant, *R* is the gas constant, *T* = 298 K, and *E*_*m*_ is the apparent midpoint potential. Fitting was performed by weighted non-linear least squares (Levenberg-Marquardt), with each point weighted by the inverse of its replicate standard deviation. The reported uncertainty on *E*_*m*_ is the standard error from the fit covariance matrix. A single heme switching between its oxidized and reduced forms produces a titration curve of a fixed width.

To estimate how many redox species account for the measured titration behavior, the oxidized-fraction data were fit to the sum of *n* independent one-electron transitions (*n* = 1–8), each with its own midpoint potential. Because a fit with many adjustable potentials can settle into a poor solution that depends on the initial parameters of the optimization, the starting values for each fit were found by differential evolution, which is a global search that tests many candidate solutions spread across the parameter space rather than refining a single initial guess (93). Adding more components always lowers the fit error, even when the extra components account only for noise, so the correct number cannot be determined by goodness-of-fit alone. The value of *n* was, therefore, chosen using information criteria. Scoring rules, the Akaike and Bayesian information criteria, which reward a closer fit but subtract a penalty for every extra fitted parameter, were used to determine the simplest model consistent with the data (94–96). Two further checks were applied. The first was a partial *F*-test, which asks whether adding one more component improves the fit more than would be expected from noise alone. The second was a residual bootstrap, which repeatedly resamples the small scatter left over between the data and the fit so that the spread of answers shows how reproducibly the same number of components is recovered (97). The number of components that can be resolved is ultimately capped by the 25 mV spacing of the potential steps and the noise of the applied potential (~1.5 mV). Finally, as a measure of the breadth of the transition that assumes no particular number of components, a Hill coefficient was obtained from the slope of log_10_([reduced]/[oxidized]) versus potential across the redox-active window of the titration curve, 0.12 < *f*_ox_ < 0.88.

## Data Availability

The three-dimensional reconstruction generated in this study has been deposited in the Electron Microscopy Data Bank under accession code EMD-73506. The atomic model generated in this study has been deposited in the Protein Data Bank under accession code 9YUQ.

## SUPPLEMENTAL MATERIAL

Supplemental material mbio.00906-26-s0001.docx Supplemental figures and table.

ASM does not own the copyrights to Supplemental Material that may be linked to, or accessed through, an article. The authors have granted ASM a non-exclusive, world-wide license to publish the Supplemental Material files. Please contact the corresponding author directly for reuse.

## References

[1] Lovley DR, Holmes DE. 2022. Electromicrobiology: the ecophysiology of phylogenetically diverse electroactive microorganisms. Nat Rev Microbiol 20:5–19. doi:10.1038/s41579-021-00597-6 34316046

[2] Shi L, Dong HL, Reguera G, Beyenal H, Lu AH, Liu J, Yu HQ, Fredrickson JK. 2016. Extracellular electron transfer mechanisms between microorganisms and minerals. Nat Rev Microbiol 14:651–662. doi:10.1038/nrmicro.2016.93 27573579

[3] Myers CR, Nealson KH. 1988. Bacterial manganese reduction and growth with manganese oxide as the sole electron acceptor. Science 240:1319–1321. doi:10.1126/science.240.4857.1319 17815852

[4] Lovley DR, Phillips EJ. 1988. Novel mode of microbial energy metabolism: organic carbon oxidation coupled to dissimilatory reduction of iron or manganese. Appl Environ Microbiol 54:1472–1480. doi:10.1128/aem.54.6.1472-1480.1988 PMC202682 16347658

[5] Summers ZM, Fogarty HE, Leang C, Franks AE, Malvankar NS, Lovley DR. 2010. Direct exchange of electrons within aggregates of an evolved syntrophic coculture of anaerobic bacteria. Science 330:1413–1415. doi:10.1126/science.1196526 21127257

[6] Reguera G. 2018. Microbial nanowires and electroactive biofilms. FEMS Microbiol Ecol 94:fiy086. doi:10.1093/femsec/fiy086 29931163

[7] Edwards MJ, Richardson DJ, Paquete CM, Clarke TA. 2020. Role of multiheme cytochromes involved in extracellular anaerobic respiration in bacteria. Protein Sci 29:830–842. doi:10.1002/pro.3787 PMC7096707 31721352

[8] Joshi K, Chan CH, Bond DR. 2021. *Geobacter sulfurreducens* inner membrane cytochrome CbcBA controls electron transfer and growth yield near the energetic limit of respiration. Mol Microbiol 116:1124–1139. doi:10.1111/mmi.14801 34423503

[9] Ouboter HT, Mesman R, Sleutels T, Postma J, Wissink M, Jetten MSM, Ter Heijne A, Berben T, Welte CU. 2024. Mechanisms of extracellular electron transfer in anaerobic methanotrophic archaea. Nat Commun 15:1477. doi:10.1038/s41467-024-45758-2 PMC10874420 38368447

[10] Pirbadian S, Barchinger SE, Leung KM, Byun HS, Jangir Y, Bouhenni RA, Reed SB, Romine MF, Saffarini DA, Shi L, Gorby YA, Golbeck JH, El-Naggar MY. 2014. *Shewanella oneidensis* MR-1 nanowires are outer membrane and periplasmic extensions of the extracellular electron transport components. Proc Natl Acad Sci USA 111:12883–12888. doi:10.1073/pnas.1410551111 PMC4156777 25143589

[11] Marsili E, Baron DB, Shikhare ID, Coursolle D, Gralnick JA, Bond DR. 2008. *Shewanella* secretes flavins that mediate extracellular electron transfer. Proc Natl Acad Sci USA 105:3968–3973. doi:10.1073/pnas.0710525105 PMC2268775 18316736

[12] Subramanian P, Pirbadian S, El-Naggar MY, Jensen GJ. 2018. Ultrastructure of MR-1 nanowires revealed by electron cryotomography. Proc Natl Acad Sci USA 115:E3246–E3255. doi:10.1073/pnas.1718810115 PMC5889646 29555764

[13] Liu Y, Wang Z, Liu J, Levar C, Edwards MJ, Babauta JT, Kennedy DW, Shi Z, Beyenal H, Bond DR, et al.. 2014. A trans-outer membrane porin-cytochrome protein complex for extracellular electron transfer by *Geobacter sulfurreducens* PCA. Environ Microbiol Rep 6:776–785. doi:10.1111/1758-2229.12204 PMC4282303 25139405

[14] Hartshorne RS, Reardon CL, Ross D, Nuester J, Clarke TA, Gates AJ, Mills PC, Fredrickson JK, Zachara JM, Shi L, Beliaev AS, Marshall MJ, Tien M, Brantley S, Butt JN, Richardson DJ. 2009. Characterization of an electron conduit between bacteria and the extracellular environment. Proc Natl Acad Sci USA 106:22169–22174. doi:10.1073/pnas.0900086106 PMC2799772 20018742

[15] Babauta J, Renslow R, Lewandowski Z, Beyenal H. 2012. Electrochemically active biofilms: facts and fiction. A review. Biofouling 28:789–812. doi:10.1080/08927014.2012.710324 PMC4242416 22856464

[16] Wang F, Craig L, Liu X, Rensing C, Egelman EH. 2023. Microbial nanowires: type IV pili or cytochrome filaments? Trends Microbiol 31:384–392. doi:10.1016/j.tim.2022.11.004 PMC10033339 36446702

[17] Wang F, Mustafa K, Suciu V, Joshi K, Chan CH, Choi S, Su Z, Si D, Hochbaum AI, Egelman EH, Bond DR. 2022. Cryo-EM structure of an extracellular Geobacter OmcE cytochrome filament reveals tetrahaem packing. Nat Microbiol 7:1291–1300. doi:10.1038/s41564-022-01159-z PMC9357133 35798889

[18] Wang F, Chan CH, Suciu V, Mustafa K, Ammend M, Si D, Hochbaum AI, Egelman EH, Bond DR. 2022. Structure of Geobacter OmcZ filaments suggests extracellular cytochrome polymers evolved independently multiple times. eLife 11:e81551. doi:10.7554/eLife.81551 PMC9473688 36062910

[19] Wang F, Gu Y, O’Brien JP, Yi SM, Yalcin SE, Srikanth V, Shen C, Vu D, Ing NL, Hochbaum AI, Egelman EH, Malvankar NS. 2019. Structure of microbial nanowires reveals stacked hemes that transport electrons over micrometers. Cell 177:361–369. doi:10.1016/j.cell.2019.03.029 PMC6720112 30951668

[20] Gu Y, Guberman-Pfeffer MJ, Srikanth V, Shen C, Giska F, Gupta K, Londer Y, Samatey FA, Batista VS, Malvankar NS. 2023. Structure of Geobacter cytochrome OmcZ identifies mechanism of nanowire assembly and conductivity. Nat Microbiol 8:284–298. doi:10.1038/s41564-022-01315-5 PMC9999484 36732469

[21] Filman DJ, Marino SF, Ward JE, Yang L, Mester Z, Bullitt E, Lovley DR, Strauss M. 2019. Cryo-EM reveals the structural basis of long-range electron transport in a cytochrome-based bacterial nanowire. Commun Biol 2:219. doi:10.1038/s42003-019-0448-9 PMC6584659 31240257

[22] Coppi MV, Leang C, Sandler SJ, Lovley DR. 2001. Development of a genetic system for Geobacter sulfurreducens. Appl Environ Microbiol 67:3180–3187. doi:10.1128/AEM.67.7.3180-3187.2001 PMC92998 11425739

[23] Chan Chi Ho, Levar CE, Jiménez-Otero F, Bond DR. 2017. Genome scale mutational analysis of *Geobacter sulfurreducens* reveals distinct molecular mechanisms for respiration and sensing of poised electrodes versus Fe(III) oxides. J Bacteriol 199:e00340-17. doi:10.1128/JB.00340-17 PMC5585712 28674067

[24] Chan C.H., Levar CE, Zacharoff L, Badalamenti JP, Bond DR. 2015. Scarless genome editing and stable inducible expression vectors for *Geobacter sulfurreducens*. Appl Environ Microbiol 81:7178–7186. doi:10.1128/AEM.01967-15 PMC4579418 26253675

[25] Petersen HA, Hochbaum AI, Bond DR, Wang F. 2026. Cytochrome nanowires under electron microscopy. Emerg Top Life Sci 8:ETLS20240008. doi:10.1042/ETLS20240008 PMC12954529 41627063

[26] Baquero DP, Cvirkaite-Krupovic V, Hu SS, Fields JL, Liu X, Rensing C, Egelman EH, Krupovic M, Wang F. 2023. Extracellular cytochrome nanowires appear to be ubiquitous in prokaryotes. Cell 186:2853–2864. doi:10.1016/j.cell.2023.05.012 PMC10330847 37290436

[27] Franks AE, Glaven RH, Lovley DR. 2012. Real-time spatial gene expression analysis within current-producing biofilms. ChemSusChem 5:1092–1098. doi:10.1002/cssc.201100714 22577044

[28] Nevin KP, Kim B-C, Glaven RH, Johnson JP, Woodard TL, Methé BA, Didonato RJ, Covalla SF, Franks AE, Liu A, Lovley DR. 2009. Anode biofilm transcriptomics reveals outer surface components essential for high density current production in *Geobacter sulfurreducens* fuel cells. PLoS One 4:e5628. doi:10.1371/journal.pone.0005628 PMC2680965 19461962

[29] Peng L, Zhang Y. 2017. Cytochrome OmcZ is essential for the current generation by *Geobacter sulfurreducens* under low electrode potential. Electrochim Acta 228:447–452. doi:10.1016/j.electacta.2017.01.091

[30] Richter H, Nevin KP, Jia HF, Lowy DA, Lovley DR, Tender LM. 2009. Cyclic voltammetry of biofilms of wild type and mutant *Geobacter sulfurreducens* on fuel cell anodes indicates possible roles of OmcB, OmcZ, type IV pili, and protons in extracellular electron transfer. Energy Environ Sci 2:506. doi:10.1039/b816647a

[31] Hall-Stoodley L, Costerton JW, Stoodley P. 2004. Bacterial biofilms: from the natural environment to infectious diseases. Nat Rev Microbiol 2:95–108. doi:10.1038/nrmicro821 15040259

[32] Vidakovic L, Mikhaleva S, Jeckel H, Nisnevich V, Strenger K, Neuhaus K, Raveendran K, Ben-Moshe NB, Aznaourova M, Nosho K, Drescher A, Schmeck B, Schulte LN, Persat A, Avraham R, Drescher K. 2023. Biofilm formation on human immune cells is a multicellular predation strategy of *Vibrio cholerae*. Cell 186:2690–2704. doi:10.1016/j.cell.2023.05.008 PMC10256282 37295405

[33] Drame I, Lafforgue C, Formosa-Dague C, Chapot-Chartier MP, Piard JC, Castelain M, Dague E. 2021. Pili and other surface proteins influence the structure and the nanomechanical properties of *Lactococcus lactis* biofilms. Sci Rep 11:4846. doi:10.1038/s41598-021-84030-1 PMC7921122 33649417

[34] Lemon KP, Higgins DE, Kolter R. 2007. Flagellar motility is critical for biofilm formation. J Bacteriol 189:4418–4424. doi:10.1128/JB.01967-06 PMC1913361 17416647

[35] Ligthart K, Belzer C, de Vos WM, Tytgat HLP. 2020. Bridging bacteria and the gut: functional aspects of type IV Pili. Trends Microbiol 28:340–348. doi:10.1016/j.tim.2020.02.003 32298612

[36] Wang F, Cvirkaite-Krupovic V, Krupovic M, Egelman EH. 2022. Archaeal bundling pili of *Pyrobaculum calidifontis* reveal similarities between archaeal and bacterial biofilms. Proc Natl Acad Sci USA 119:e2207037119. doi:10.1073/pnas.2207037119 PMC9245690 35727984

[37] Böhning J, Ghrayeb M, Pedebos C, Abbas DK, Khalid S, Chai L, Bharat TAM. 2022. Donor-strand exchange drives assembly of the TasA scaffold in *Bacillus subtilis* biofilms. Nat Commun 13:7082. doi:10.1038/s41467-022-34700-z PMC9674648 36400765

[38] Böhning J, Dobbelstein AW, Sulkowski N, Eilers K, von Kügelgen A, Tarafder AK, Peak-Chew S-Y, Skehel M, Alva V, Filloux A, Bharat TAM. 2023. Architecture of the biofilm-associated archaic Chaperone-Usher pilus CupE from *Pseudomonas aeruginosa**.* PLoS Pathog 19:e1011177. doi:10.1371/journal.ppat.1011177 PMC10104325 37058467

[39] Fields JL, Sebastian CC, Zhang H, Zia A, Robertson AN, Wu H, Kiedrowski MR, Wang F. 2026. The archaic CUP pilus SMF-1 utilizes a specific antiparallel bundling mechanism to initiate biofilm formation. bioRxiv:2026.02.19.706768. doi:10.64898/2026.02.19.70676842826101

[40] Malmi H, Pakharukova N, Paul B, Tuittila M, Ahmad I, Knight SD, Uhlin BE, Ghosal D, Zavialov AV. 2026. Antiparallel stacking of Csu pili drives *Acinetobacter baumannii* 3D biofilm assembly. Nat Commun 17. doi:10.1038/s41467-026-68860-z PMC12996565 41654547

[41] Doucey MA, Carrara S. 2019. Nanowire sensors in cancer. Trends Biotechnol 37:86–99. doi:10.1016/j.tibtech.2018.07.014 30126620

[42] Wang Z, Lee S, Koo K, Kim K. 2016. Nanowire-based sensors for biological and medical applications. IEEE Trans Nanobioscience 15:186–199. doi:10.1109/TNB.2016.2528258 26978831

[43] Domigan LJ. 2013. Proteins and peptides as biological nanowires: towards biosensing devices, p 131–152. *In* Protein nanotechnology: protocols, instrumentation, and applications. Humana Press. 10.1007/978-1-62703-354-1_8 23504422

[44] Abramson J, Adler J, Dunger J, Evans R, Green T, Pritzel A, Ronneberger O, Willmore L, Ballard AJ, Bambrick J, et al.. 2024. Accurate structure prediction of biomolecular interactions with AlphaFold 3. Nature 630:493–500. doi:10.1038/s41586-024-07487-w PMC11168924 38718835

[45] Badalamenti JP, Summers ZM, Chan CH, Gralnick JA, Bond DR. 2016. Isolation and genomic characterization of “*Desulfuromonas soudanensis* WTL”, a metal- and electrode-respiring bacterium from anoxic deep subsurface brine. Front Microbiol 7:913. doi:10.3389/fmicb.2016.00913 PMC4914508 27445996

[46] Wang F, Coureuil M, Osinski T, Orlova A, Altindal T, Gesbert G, Nassif X, Egelman EH, Craig L. 2017. Cryoelectron microscopy reconstructions of the *Pseudomonas aeruginosa* and *Neisseria gonorrhoeae* type IV pili at sub-nanometer resolution. Structure 25:1423–1435. doi:10.1016/j.str.2017.07.016 PMC8189185 28877506

[47] Bazayeva M, Andreini C, Rosato A. 2024. A database overview of metal-coordination distances in metalloproteins. Acta Crystallogr D Struct Biol 80:362–376. doi:10.1107/S2059798324003152 PMC11066882 38682667

[48] Krissinel E, Henrick K. 2007. Inference of macromolecular assemblies from crystalline state. J Mol Biol 372:774–797. doi:10.1016/j.jmb.2007.05.022 17681537

[49] Tsapin AI, Vandenberghe I, Nealson KH, Scott JH, Meyer TE, Cusanovich MA, Harada E, Kaizu T, Akutsu H, Leys D, Van Beeumen JJ. 2001. Identification of a small tetraheme cytochrome c and a flavocytochrome c as two of the principal soluble cytochromes c in *Shewanella oneidensis* strain MR1. Appl Environ Microbiol 67:3236–3244. doi:10.1128/AEM.67.7.3236-3244.2001 PMC93006 11425747

[50] Lommen A, Ratsma A, Bijlsma N, Canters GW, van Wielink JE, Frank J, van Beeumen J. 1990. Isolation and characterization of cytochrome c550 from the methylamine-oxidizing electron-transport chain of *Thiobacillus versutus*. Eur J Biochem 192:653–661. doi:10.1111/j.1432-1033.1990.tb19272.x 2170127

[51] Van Gelder BF, Slater EC. 1962. The extinction coefficient of cytochrome c. Biochim Biophys Acta 58:593–595. doi:10.1016/0006-3002(62)90073-2 13897582

[52] Portela PC, Shipps CC, Shen C, Srikanth V, Salgueiro CA, Malvankar NS. 2024. Widespread extracellular electron transfer pathways for charging microbial cytochrome OmcS nanowires via periplasmic cytochromes PpcABCDE. Nat Commun 15:2434. doi:10.1038/s41467-024-46192-0 PMC10954620 38509081

[53] Hardy BJ, Dubiel P, Bungay EL, Rudin M, Williams C, Arthur CJ, Guberman-Pfeffer MJ, Sofia Oliveira A, Curnow P, Anderson JLR. 2024. Delineating redox cooperativity in water-soluble and membrane multiheme cytochromes through protein design. Protein Sci 33:e5113. doi:10.1002/pro.5113 PMC11232281 38980168

[54] Inoue K, Qian X, Morgado L, Kim B-C, Mester T, Izallalen M, Salgueiro CA, Lovley DR. 2010. Purification and characterization of OmcZ, an outer-surface, octaheme c-type cytochrome essential for optimal current production by *Geobacter sulfurreducens*. Appl Environ Microbiol 76:3999–4007. doi:10.1128/AEM.00027-10 PMC2893489 20400562

[55] Santos TC, Silva MA, Morgado L, Dantas JM, Salgueiro CA. 2015. Diving into the redox properties of *Geobacter sulfurreducens* cytochromes: a model for extracellular electron transfer. Dalton Trans 44:9335–9344. doi:10.1039/c5dt00556f 25906375

[56] Morgado L, Bruix M, Orshonsky V, Londer YY, Duke NEC, Yang X, Pokkuluri PR, Schiffer M, Salgueiro CA. 2008. Structural insights into the modulation of the redox properties of two *Geobacter sulfurreducens* homologous triheme cytochromes. Biochim Biophys Acta Bioenerget 1777:1157–1165. doi:10.1016/j.bbabio.2008.04.043 18534185

[57] Childers SE, Ciufo S, Lovley DR. 2002. *Geobacter metallireducens* accesses insoluble Fe(III) oxide by chemotaxis. Nature 416:767–769. doi:10.1038/416767a 11961561

[58] Rotaru AE, Shrestha PM, Liu F, Markovaite B, Chen S, Nevin KP, Lovley DR. 2014. Direct interspecies electron transfer between *Geobacter metallireducens* and *Methanosarcina barkeri*. Appl Environ Microbiol 80:4599–4605. doi:10.1128/AEM.00895-14 PMC4148795 24837373

[59] Kühlbrandt W. 2014. Biochemistry. The resolution revolution. Science 343:1443–1444. doi:10.1126/science.1251652 24675944

[60] Jamali K, Käll L, Zhang R, Brown A, Kimanius D, Scheres SHW. 2024. Automated model building and protein identification in cryo-EM maps. Nature 628:450–457. doi:10.1038/s41586-024-07215-4 PMC11006616 38408488

[61] Pfab J, Phan NM, Si D. 2021. DeepTracer for fast *de novo* cryo-EM protein structure modeling and special studies on CoV-related complexes. Proc Natl Acad Sci USA 118:e2017525118. doi:10.1073/pnas.2017525118 PMC7812826 33361332

[62] Wang T, Li Z, Xu K, Huang W, Huang G, Zhang QC, Yan N. 2024. CryoSeek: A strategy for bioentity discovery using cryoelectron microscopy. Proc Natl Acad Sci USA 121:e2417046121. doi:10.1073/pnas.2417046121 PMC11494351 39382995

[63] Semchonok DA, Kyrilis FL, Hamdi F, Kastritis PL. 2023. Cryo-EM of a heterogeneous biochemical fraction elucidates multiple protein complexes from a multicellular thermophilic eukaryote. J Struct Biol X 8:100094. doi:10.1016/j.yjsbx.2023.100094 PMC10451023 37638207

[64] Liu J, Eastep GN, Cvirkaite-Krupovic V, Rich-New ST, Kreutzberger MAB, Egelman EH, Krupovic M, Wang F. 2024. Two distinct archaeal type IV pili structures formed by proteins with identical sequence. Nat Commun 15:5049. doi:10.1038/s41467-024-45062-z PMC11178852 38877064

[65] Voica DM, Bartha L, Banciu HL, Oren A. 2016. Heavy metal resistance in halophilic Bacteria and Archaea. FEMS Microbiol Lett 363:fnw146. doi:10.1093/femsle/fnw146 27279625

[66] Nies DH. 2003. Efflux-mediated heavy metal resistance in prokaryotes. FEMS Microbiol Rev 27:313–339. doi:10.1016/S0168-6445(03)00048-2 12829273

[67] Liu J, Chakraborty S, Hosseinzadeh P, Yu Y, Tian SL, Petrik I, Bhagi A, Lu Y. 2014. Metalloproteins containing cytochrome, iron-sulfur, or copper redox centers. Chem Rev 114:4366–4469. doi:10.1021/cr400479b PMC4002152 24758379

[68] Pakharukova N, Malmi H, Tuittila M, Dahlberg T, Ghosal D, Chang Y-W, Myint SL, Paavilainen S, Knight SD, Lamminmäki U, Uhlin BE, Andersson M, Jensen G, Zavialov AV. 2022. Archaic chaperone-usher pili self-secrete into superelastic zigzag springs. Nature 609:335–340. doi:10.1038/s41586-022-05095-0 PMC9452303 35853476

[69] Nuccio S-P, Bäumler AJ. 2007. Evolution of the chaperone/usher assembly pathway: fimbrial classification goes Greek. Microbiol Mol Biol Rev 71:551–575. doi:10.1128/MMBR.00014-07 PMC2168650 18063717

[70] Vallet I, Olson JW, Lory S, Lazdunski A, Filloux A. 2001. The chaperone/usher pathways of *Pseudomonas aeruginosa*: identification of fimbrial gene clusters (cup) and their involvement in biofilm formation. Proc Natl Acad Sci USA 98:6911–6916. doi:10.1073/pnas.111551898 PMC34452 11381121

[71] Gu Y, Srikanth V, Salazar-Morales AI, Jain R, O’Brien JP, Yi SM, Soni RK, Samatey FA, Yalcin SE, Malvankar NS. 2021. Structure of *Geobacter pili* reveals secretory rather than nanowire behaviour. Nature 597:430–434. doi:10.1038/s41586-021-03857-w PMC9127704 34471289

[72] Shen C, Salazar-Morales AI, Jung W, Erwin J, Gu Y, Coelho A, Gupta K, Yalcin SE, Samatey FA, Malvankar NS. 2025. A widespread and ancient bacterial machinery assembles cytochrome OmcS nanowires essential for extracellular electron transfer. Cell Chem Biol 32:239–254. doi:10.1016/j.chembiol.2024.12.013 PMC11845295 39818215

[73] Allen WJ, Phan G, Waksman G. 2012. Pilus biogenesis at the outer membrane of Gram-negative bacterial pathogens. Curr Opin Struct Biol 22:500–506. doi:10.1016/j.sbi.2012.02.001 22402496

[74] Werneburg GT, Thanassi DG. 2018. Pili assembled by the chaperone/usher pathway in *Escherichia coli* and *Salmonella*. EcoSal Plus 8. doi:10.1128/ecosalplus.ESP-0007-2017 PMC5940347 29536829

[75] Busch A, Waksman G. 2012. Chaperone–usher pathways: diversity and pilus assembly mechanism. Phil Trans R Soc B 367:1112–1122. doi:10.1098/rstb.2011.0206 PMC3297437 22411982

[76] Zheng SQ, Palovcak E, Armache JP, Verba KA, Cheng Y, Agard DA. 2017. MotionCor2: anisotropic correction of beam-induced motion for improved cryo-electron microscopy. Nat Methods 14:331–332. doi:10.1038/nmeth.4193 PMC5494038 28250466

[77] Punjani A, Rubinstein JL, Fleet DJ, Brubaker MA. 2017. cryoSPARC: algorithms for rapid unsupervised cryo-EM structure determination. Nat Methods 14:290–296. doi:10.1038/nmeth.4169 28165473

[78] Rohou A, Grigorieff N. 2015. CTFFIND4: fast and accurate defocus estimation from electron micrographs. J Struct Biol 192:216–221. doi:10.1016/j.jsb.2015.08.008 PMC6760662 26278980

[79] Wang F, Gnewou O, Solemanifar A, Conticello VP, Egelman EH. 2022. Cryo-EM of helical polymers. Chem Rev 122:14055–14065. doi:10.1021/acs.chemrev.1c00753 PMC9357864 35133794

[80] Pettersen EF, Goddard TD, Huang CC, Meng EC, Couch GS, Croll TI, Morris JH, Ferrin TE. 2021. UCSF ChimeraX: structure visualization for researchers, educators, and developers. Protein Sci 30:70–82. doi:10.1002/pro.3943 PMC7737788 32881101

[81] Sanchez-Garcia R, Gomez-Blanco J, Cuervo A, Carazo JM, Sorzano COS, Vargas J. 2021. DeepEMhancer: a deep learning solution for cryo-EM volume post-processing. Commun Biol 4:874. doi:10.1038/s42003-021-02399-1 PMC8282847 34267316

[82] Wang RY-R, Kudryashev M, Li X, Egelman EH, Basler M, Cheng Y, Baker D, DiMaio F. 2015. *De novo* protein structure determination from near-atomic-resolution cryo-EM maps. Nat Methods 12:335–338. doi:10.1038/nmeth.3287 PMC4435692 25707029

[83] Clarke TA, Cole JA, Richardson DJ, Hemmings AM. 2007. The crystal structure of the pentahaem c-type cytochrome NrfB and characterization of its solution-state interaction with the pentahaem nitrite reductase NrfA. Biochem J 406:19–30. doi:10.1042/BJ20070321 PMC1948984 17521287

[84] Rodrigues ML, Oliveira TF, Pereira IAC, Archer M. 2006. X-ray structure of the membrane-bound cytochrome c quinol dehydrogenase NrfH reveals novel haem coordination. EMBO J 25:5951–5960. doi:10.1038/sj.emboj.7601439 PMC1698886 17139260

[85] Emsley P, Cowtan K. 2004. Coot: model-building tools for molecular graphics. Acta Crystallogr D Biol Crystallogr 60:2126–2132. doi:10.1107/S0907444904019158 15572765

[86] Afonine PV, Poon BK, Read RJ, Sobolev OV, Terwilliger TC, Urzhumtsev A, Adams PD. 2018. Real-space refinement in PHENIX for cryo-EM and crystallography. Acta Crystallogr D Struct Biol 74:531–544. doi:10.1107/S2059798318006551 PMC6096492 29872004

[87] Williams CJ, Headd JJ, Moriarty NW, Prisant MG, Videau LL, Deis LN, Verma V, Keedy DA, Hintze BJ, Chen VB, Jain S, Lewis SM, Arendall WB 3rd, Snoeyink J, Adams PD, Lovell SC, Richardson JS, Richardson DC. 2018. MolProbity: more and better reference data for improved all-atom structure validation. Protein Sci 27:293–315. doi:10.1002/pro.3330 PMC5734394 29067766

[88] Qian X, Mester T, Morgado L, Arakawa T, Sharma ML, Inoue K, Joseph C, Salgueiro CA, Maroney MJ, Lovley DR. 2011. Biochemical characterization of purified OmcS, a c-type cytochrome required for insoluble Fe(III) reduction in *Geobacter sulfurreducens*. Biochim Biophys Acta Bioenerget 1807:404–412. doi:10.1016/j.bbabio.2011.01.003 21236241

[89] Berry EA, Trumpower BL. 1987. Simultaneous determination of hemes a, b, and c from pyridine hemochrome spectra. Anal Biochem 161:1–15. doi:10.1016/0003-2697(87)90643-9 3578775

[90] Harris CR, Millman KJ, van der Walt SJ, Gommers R, Virtanen P, Cournapeau D, Wieser E, Taylor J, Berg S, Smith NJ, et al.. 2020. Array programming with NumPy. Nature 585:357–362. doi:10.1038/s41586-020-2649-2 PMC7759461 32939066

[91] Basalla AJ, Kendrick BS. 2023. Correcting ultraviolet-visible spectra for baseline artifacts. J Pharm Sci 112:3240–3247. doi:10.1016/j.xphs.2023.08.015 37619816

[92] Savitzky Abraham, Golay MJE. 1964. Smoothing and differentiation of data by simplified least squares procedures. Anal Chem 36:1627–1639. doi:10.1021/ac60214a047 22324618

[93] Storn R, Price K. 1997. Differential evolution – a simple and efficient heuristic for global optimization over continuous spaces. J Glob Optim 11:341–359. doi:10.1023/A:1008202821328

[94] Burnham KP, Anderson DR. 2004. Multimodel inference: understanding AIC and BIC in model selection. Sociol Methods Res 33:261–304. doi:10.1177/0049124104268644

[95] Schwarz G. 1978. Estimating the dimension of a model. Ann Statist 6. doi:10.1214/aos/1176344136

[96] Akaike H. 1974. A new look at the statistical model identification. IEEE Trans Automat Contr 19:716–723. doi:10.1109/TAC.1974.1100705

[97] Efron B. 1979. Bootstrap methods: another look at the Jackknife. Ann Statist 7:1–26. doi:10.1214/aos/1176344552

