# Supplementary material for: A bundled anti-parallel cytochrome nanowire structure suggests roles in cell-cell electron transfer and biofilm formation
Source: mBio. 2026 Aug 26;17(10):e00906-26. doi: 10.1128/mbio.00906-26 (PMC13712944; doi:10.1128/mbio.00906-26)
Supplement: Supplementary file 1 [file mbio.00906-26-s0001.docx]

**Supplementary Table 1. Cryo-EM and refinement statistics of *Desulfuromonas soudanensis* WTL OmcE-like cytochrome nanowire**

| **Parameter** |  |
| --- | --- |
| **Data collection and processing** | |
| Voltage (kV) | 300 |
| Electron exposure (e^−^ Å^−2^) | 50 |
| Pixel size (Å) | 1.07 |
| Particle images (n) | 1,241,521 |
| Shift (pixel) | 30 |
| **Helical symmetry,** single filament | |
| Point group | C1 |
| Helical rise (Å) | 30.76 |
| Helical twist (°) | 51.44 |
| **Map resolution (Å)**, asymmetrical reconstruction | |
| Map:map FSC (0.143) | 3.1 |
| Model:map FSC (0.5) | 3.4 |
| **Refinement and Model validation** | |
| Ramachandran Favored (%) | 88.7 |
| Ramachandran Outliers (%) | 0.4 |
| RSCC | 0.76 |
| Clashscore | 22.2 |
| Bonds RMSD, length (Å) | 0.006 |
| Bonds RMSD, angles (°) | 1.399 |
| **Deposition ID** |  |
| PDB (model) | 9YUQ |
| EMDB (map) | EMD-73506 |

**Supplementary Figure 1. *Sheared D. soudanensis nanowires in different buffers.***

To ensure that the formed cytochrome nanowire bundles are not an artifact of buffer conditions, we examined the bundles in three different buffer conditions: phosphate-buffered saline (left), bacterial high-salt growth medium (middle), and 10 mM ethanolamine buffer, pH 10.5 (right). The scale bar in the medium magnification is 1 µm, and the scale bars in the high magnification are 100 nm. The bundles are indicated with yellow arrowheads.

**Supplementary Figure 2. Average Power Spectrum and Fourier Shell Correlation (FSC) Calculations**

**A**. The average power spectrum of nanowire raw particles aligned to the same axis.

**B**. The map-to-map FSC calculation of the helical reconstruction of *D. soudanensis* cytochrome nanowire (0.143 cutoff).

**C**. The map-to-map FSC calculation of the asymmetrical reconstruction of *D. soudanensis* cytochrome nanowire bundles (0.143 cutoff).

**Supplementary Figure 3. Tilted cryo-EM data collection to overcome preferred orientation in OmcE bundles**

**A**. Using the aircraft principal axes as an analogy to illustrate tilted data collection on filament bundles, a tilted grid can be viewed as having a combination of azimuthal rotation and an out-of-plane tilt.

**B**. Initial cryo-EM analysis of non-tilted datasets revealed that the cytochrome bundles adopt a preferred orientation in ice, yielding only seven distinct views. This is largely because these OmcE bundles form a flat network and the helical twist of the cytochrome subunits within a single nanowire is 51.4°, which is very close to 360° divided by 7. The right panel illustrates two extreme cases encountered during tilted data collection. Tilt axis 1 is parallel to the helical axis of a single filament; therefore, a bundle particle in this orientation will generate images with a 12-degree azimuthal rotation and no out-of-plane tilt. Tilt axis 2, on the other hand, is perpendicular to the helical axis. If a bundle particle is oriented in this direction, the resulting image will have the same azimuthal rotation as an untitled particle but with an extra 12-degree out-of-plane tilt. In practice, all particles will be oriented somewhere between tilt axis 1 and axis 2, thereby covering the entire 12-degree azimuthal rotation range.

**C**. Cryo-EM collects 2D projections of a 3D object. Consequently, for any particle image A, rotating the bundle structure by 180° along the helical axis will generate a mirror particle of image A, which is thus identical to image A during data processing. We can therefore plot the covered azimuthal views from 0 to 180 degrees for the cytochrome bundles. By collecting data on grids with 12 degree tilts, we obtained particles covering most azimuthal views as indicated.

**Supplementary Figure 4. Ion Binding Sites in Cytochrome Nanowire Bundles**

**A**. Ribbon view of the cytochrome nanowire bundles, with adjacent protein subunits colored differently. Putative calcium ions are displayed as green spheres.

**B**. A zoomed-in view showing the putative calcium ions within the cryo-EM map. Protein backbones are shown as ribbons, while side chains are displayed as sticks.

**C**. Contacts between the putative calcium ions and nearby oxygen atoms (O-atoms) from adjacent residues or a heme c molecule. Water molecules are typically invisible at this resolution.

**Supplementary Figure 5.** **Local resolution estimation of the asymmetrical reconstruction**

Based on cryoSPARC local filtering analysis, the region with the highest resolution reaches ~2.6 Å. The majority of the map used for modeling (Figure 1) ranges between 2.6 Å and 4 Å. The filament with the best resolution, which was used to build the initial single filament model, ranges between 2.6 Å and 3.4 Å.

**Supplemental Figure 6. Determination of the *D. soudanensis* OmcE α-band extinction coefficient.**

(a) Absorbance spectra of intact native protein: oxidized (red) and dithionite-reduced (blue), with the fitted RMO baseline (dashed; fit window 600-700 nm shaded). (b) RMO-corrected native protein α-band absorbance spectra: the reduced−oxidized difference (dashed) peaks at 552 nm. (c) RMO-corrected pyridine hemochrome (oxidized vs reduced), used to quantify heme concentration.

**Supplemental Figure 7. Scattering-corrected UV-vis spectra of *D. soudanensis* OmcE nanowire bundles across the spectroelectrochemical titration.**

**Supplemental Figure 8. Number of resolvable redox components in the *D. soudanensis* OmcE titration.**

(a) Residual sum of squares of the multi-component Nernst fit versus the number of components *n*, which saturates at *n* = 5. (b) Akaike, Bayesian and corrected-Akaike information criteria versus *n*, all minimized at *n* = 5. Together these metrics suggest five resolvable redox components are electrostatically coupled at any given point along the *D. soudanensis* OmcE bundles.

**Supplementary Figure 9. Cryo-EM of Putative OmcE Bundles in *Geobacter metallireducens***

**A**. Cryo-EM micrograph of cytochrome bundles from *Geobacter metallireducens*. Bundles are indicated by yellow arrowheads. Single fibers disassociated from the bundle are indicated with blue arrowheads. Scale bar: 50 nm.

**B**. 2D averages of cytochrome bundles from *Geobacter metallireducens*.

**C**. Indexing of the average power spectrum from aligned *Geobacter metallireducens* cytochrome bundles.

**D**. The helical symmetry of the bundles in *Geobacter metallireducens*, compared to other bacterial cytochrome filaments, including other OmcE proteins, OmcS, and OmcZ.

**Supplementary Figure 10. *De novo* atomic model building of OmcE filaments in *D. soudanensis* WTL**

**A.** Sequence-based alignment of OmcE (DSOUD_2954) and two other c-type cytochromes (DSOUD_2415 and DSOUD_0663) with a similar protein fold.

**B**. and **C**. The zoomed views of one example region indicated in (**A**) by a green box, showing the atomic model of the other two OmcE-like proteins fit into the cryo-EM map. Disagreements between the map and incorrect sequences are obvious.
